# CD97 stabilises the immunological synapse between dendritic cells and T cells and is targeted for degradation by the *Salmonella* effector SteD

**DOI:** 10.1371/journal.ppat.1009771

**Published:** 2021-07-27

**Authors:** Ondrej Cerny, Camilla Godlee, Romina Tocci, Nancy E. Cross, Haoran Shi, James C. Williamson, Eric Alix, Paul J. Lehner, David W. Holden

**Affiliations:** 1 MRC Centre for Molecular Bacteriology and Infection, Imperial College London, London, United Kingdom; 2 Cambridge Institute for Therapeutic Immunology and Infectious Disease (CITIID), University of Cambridge, Cambridge, United Kingdom; University of California Davis School of Medicine, UNITED STATES

## Abstract

The *Salmonella enterica* effector SteD depletes mature MHC class II (mMHCII) molecules from the surface of infected antigen-presenting cells through ubiquitination of the cytoplasmic tail of the mMHCII β chain. This requires the Nedd4 family HECT E3 ubiquitin ligase Wwp2 and a tumor-suppressing transmembrane protein adaptor Tmem127. Here, through a proteomic screen of dendritic cells, we found that SteD targets the plasma membrane protein CD97 for degradation by a similar mechanism. SteD enhanced ubiquitination of CD97 on K555 and mutation of this residue eliminated the effect of SteD on CD97 surface levels. We showed that CD97 localises to and stabilises the immunological synapse between dendritic cells and T cells. Removal of CD97 by SteD inhibited dendritic cell-T cell interactions and reduced T cell activation, independently of its effect on MHCII. Therefore, SteD suppresses T cell immunity by two distinct processes.

## Author summary

*Salmonella enterica* is the causative agent of very large numbers of serious and life-threatening diseases in humans and livestock throughout the world. Clearance of *S*. *enterica* from the host is dependent on T cell-mediated immune responses. We show here that the *Salmonella* SPI-2 type III secretion system effector SteD inhibits activation of T cells by reducing contacts between infected antigen-presenting cells and T cells. This is mediated by degradation of an adhesion G protein-coupled receptor CD97. Our work reveals that CD97 stabilizes the interaction between antigen-presenting cells and T cells and identifies this process as a direct target for bacterial pathogens.

## Introduction

Infection and development of potentially life-threatening disease in mammals by *Salmonella enterica* depends on evasion and suppression of host immune responses. Innate immune responses are directly suppressed by several virulence proteins (effectors) delivered into host cells by *Salmonella* pathogenicity island (SPI) 1 and 2-encoded type 3 secretion systems (T3SSs) [1]. Increasing evidence shows that *Salmonella enterica* also interferes with adaptive immune responses including T cell responses [2].

Activation of T cells depends on appropriate stimulation by antigen-presenting cells, such as dendritic cells (DCs). This is driven by cell-cell contact at the immunological synapse (IS), where DCs present antigenic peptides to CD4^+^ T cells (by major histocompatibility complex class II molecules (MHCII) molecules) and CD8^+^ T cells (by MHCI molecules) along with associated co-stimulatory signals mediated, for example, by CD80 or CD86. The IS is stabilised over time by protein-protein interactions [3]. While CD8^+^ T cells contribute to anti-*Salmonella* protection in certain mouse strains [4], there is to date little evidence for their involvement in human immunity to *Salmonella*. On the other hand, CD4^+^ T cells have a major role in clearance of *Salmonella* from systemic tissues in both mice [5] and humans [6].

We showed previously that the small, transmembrane SPI-2 T3SS effector SteD decreases surface levels of MHCII on DCs [7]. Following its translocation into infected DCs, SteD forms a complex with endosomal mature MHCII (mMHCII) and the transmembrane protein Tmem127, which serves as an adaptor for Nedd4 family HECT E3 ubiquitin ligase Wwp2 [8]. This interaction leads to the ubiquitination and lysosomal degradation of mMHCII, which prevents activation of CD4^+^ T cells [8]. SteD also depletes cell surface levels of CD86 [7], suggesting that it might have multiple targets.

To screen for additional host proteins whose cell surface abundance is affected by SteD, we enriched plasma membrane proteins through selective aminooxy-biotinylation (Plasma Membrane Profiling; PMP) [9]. This unbiased, systematic analysis identified CD97, (Adhesion G protein-coupled receptor E5; *Adgre5*), as an SteD target. We found that CD97 localises to and stabilises the DC-T cell IS. SteD-dependent ubiquitination of CD97 by Tmem127/Wwp2 led to lysosomal degradation of CD97 and reduced stable interactions between DCs and T cells. This in turn reduced activation of both CD4^+^ and CD8^+^ T cells independently of antigen presentation, thereby enabling a distinct means of immune evasion.

## Results

### Quantitative differential analysis of cell surface proteins identifies CD97 as a new target of SteD

**Fig 1:**
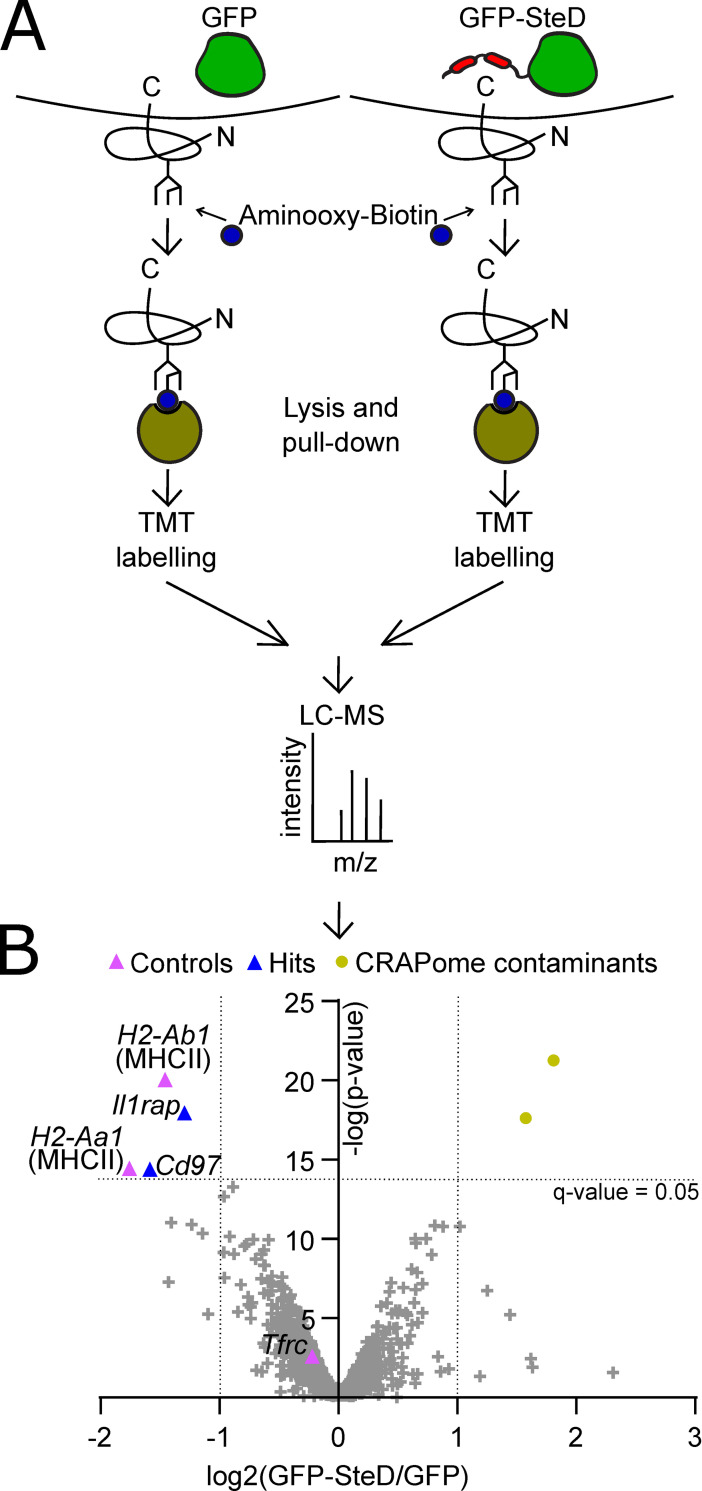
Plasma membrane proteome screens. (A) Schematic of plasma membrane glycoprotein biotinylation screen using Aminooxy-Biotin. Plasma membrane proteins biotinylated on their extracellular moieties were enriched using streptavidin beads and TMT-labelled before pooling and identification by mass spectrometry. (B) Volcano plot showing proteins in pooled samples of cells expressing GFP or GFP-SteD. log2(GFP-SteD/GFP) fold change of +/- 1 and q-value of 0.05 were chosen as thresholds to identify significantly affected proteins.

To determine if SteD influences the levels of surface proteins other than MHCII and CD86 in antigen-presenting cells, we used an unbiased proteomic approach to compare the relative abundance of plasma membrane proteins in MutuDCs, a commonly used mouse dendritic cell line [10], stably expressing GFP or GFP-SteD, after cells had been activated with LPS over the course of 48 hours. Plasma membrane proteins were selectively biotinylated by exposing intact cells to Aminooxy-biotin (reacting with N-linked sialic acid, frequently the terminal glycan residue of plasma membrane glycoproteins) (Fig 1A). Biotinylated proteins were collected at 6, 24 and 48 hours using streptavidin agarose, labelled with different isobaric chemical tags (tandem mass tags, TMT), proteolytically digested and pooled before mass-spectrometry analysis to simplify the identification of proteins that were affected in a sustained manner. This method identified 3,248 proteins from cells containing either GFP or GFP-SteD (Fig 1B and S1 Table). Of these, 998 were annotated as plasma membrane proteins by the Gene Ontology database (31%), which accounted for 56% of total protein abundance. The Contaminant Repository for Affinity Purification Mass Spectrometry Data for pull down of membranous proteins (CRAPome; [11] was used to remove proteins that were likely to represent false positives. To qualify as hits, remaining proteins had to be identified by at least 3 unique peptides, to have a false discovery rate (q-value) lower than 0.05 and a log2 fold change between cells expressing GFP or GFP-SteD of at least +/- 1.0.

Three proteins (MHCII, TfR and CD86) were used to assess the quality of the screen. Both α and β chains of the H2A isoform of MHCII, a known target of SteD, were identified as strongly depleted in biotinylated samples from SteD-expressing cells (Fig 1B and S1 Table). In contrast, the level of transferrin receptor TfR (*Tfrc*), which is not targeted by SteD [7], was not affected by the presence of SteD (Fig 1B and S1 Table). CD86, another target of SteD [7], was not identified as a hit by this method (S1 Table).

In addition to MHCII, two other proteins qualified as significant hits: Interleukin-1 receptor accessory protein (*Il1rap*) and CD97. The adhesion G protein-coupled receptor CD97 is involved in immunoregulation [12]. IL1rap associates with Interleukin-1 receptor and is necessary for formation of the receptor complex for interleukin-1β [13]. Alternative splicing of Il1rap generates both membrane-bound and soluble products, complicating its analysis [14]. We were unable to obtain antibodies specifically recognising murine Il1rap and therefore focussed on CD97.

### CD97 is targeted by SteD

**Fig 2:**
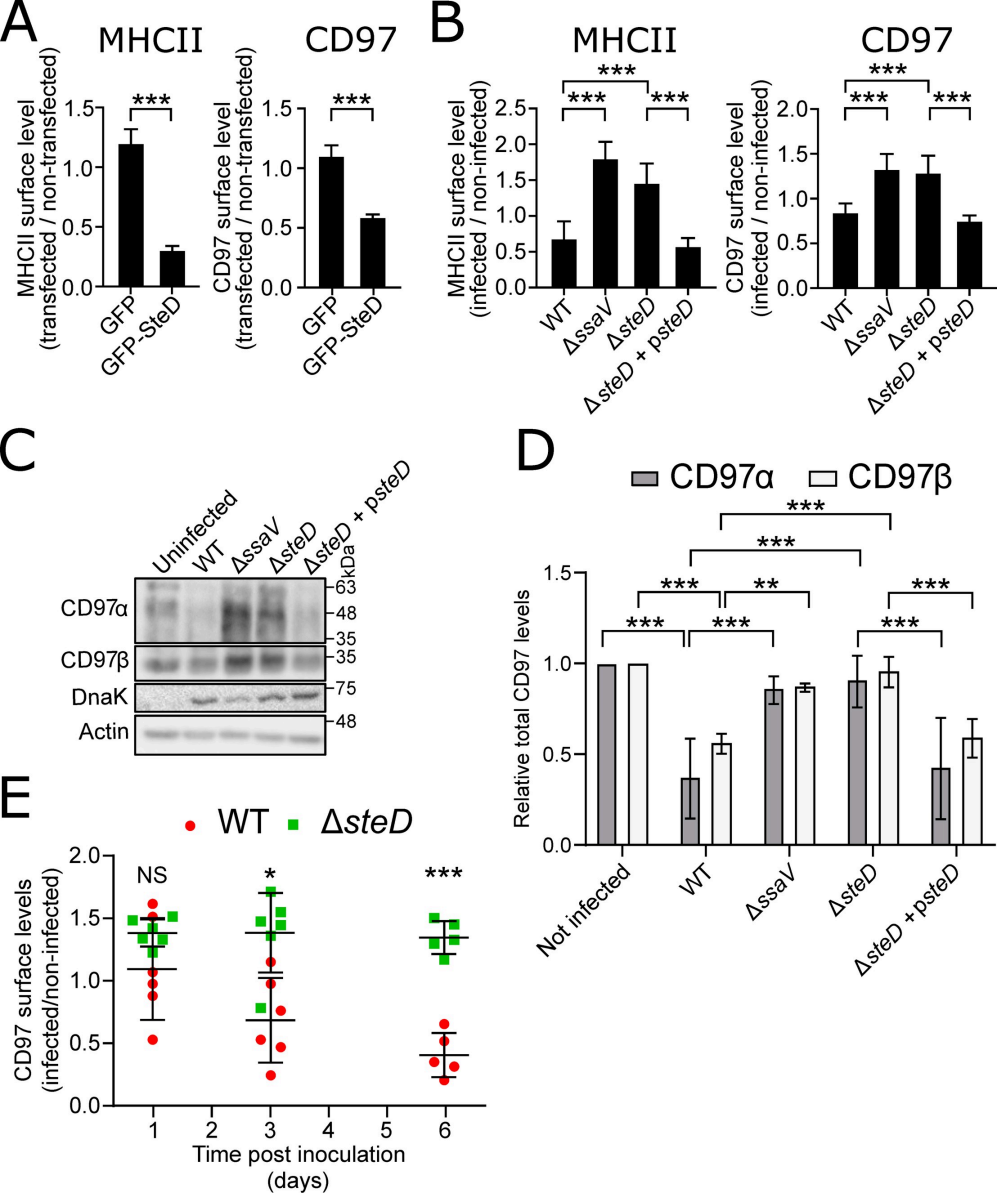
Validation of screen hits. (A) Quantification of MHCII and CD97α surface levels in MutuDCs expressing either GFP or GFP-SteD. Cells were analysed by flow cytometry and amounts of indicated proteins in transfected cells are expressed as a fraction of fluorescence of non-transfected cells in the same sample. Data are from 3 independent experiments and show means ± SD. *** p < 0.001 (Student’s T-test). (B) Quantification of MHCII and CD97α surface levels in MutuDCs infected with the indicated *S*. Typhimurium strains. Cells were analysed by flow cytometry 24 h p.i. and amounts of surface proteins in infected cells are expressed as a fraction of fluorescence of non-infected cells in the same sample. Data are from 3 independent experiments and show means ± SD. *** p < 0.001, NS—not significant (one-way ANOVA followed by Tukey’s multiple comparison test). (C) Total levels of CD97α and β subunits in whole cell lysates of MutuDCs infected with the indicated *S*. Typhimurium strains for 24 h. Samples were analysed by SDS-PAGE and immunoblot using polyclonal anti-CD97α and anti-CD97β antibodies and monoclonal anti-actin and anti-DnaK (*Salmonella*) antibodies. Representative of 3 independent experiments. (D) Quantification of intensity of CD97α and CD97β signal relative to non-infected cells, from 3 experiments represented in (C). Data are means ± SD from three independent experiments. ** p < 0.01; *** p<0.001 (two-way ANOVA followed by Tukey’s multiple comparison test). (E) Quantification of CD97α surface levels in infected dendritic cells *in vivo*. Cells were obtained from draining MLNs of C57BL/6 mice at indicated times post-oral inoculation with WT-GFP or Δ*steD*-GFP *S*. Typhimurium. CD11c^+^ cells were isolated by magnetic separation and CD97α surface levels were analysed by flow cytometry. Amounts of surface CD97α in infected cells are expressed as a fraction of fluorescence of non-infected cells in the same sample. Each dot represents the value from CD11c^+^ cells obtained from MLNs pooled from two or three mice from three independent experiments and means ± SD are shown. * p < 0.05, *** p < 0.001, NS—not significant (two-way ANOVA followed by Tukey’s multiple comparison test).

To examine CD97 in greater detail, we analysed surface levels of MHCII and CD97 in MutuDCs stably expressing GFP or GFP-SteD by flow cytometry using specific antibodies. As expected, a substantial portion of MHCII was removed from the surface of MutuDCs containing SteD (Figs 2A and S1A). Similarly, ectopic expression of SteD significantly reduced surface CD97 from MutuDCs, confirming the results of the proteomic screens (Figs 2A and S1A).

To evaluate the effect of physiological levels of SteD on surface levels of CD97, MutuDCs were examined by flow cytometry after infection with either wild type (WT) *S*. Typhimurium, a SPI-2 T3SS mutant (Δ*ssaV;* unable to translocate effectors through the SPI-2 T3SS), an Δ*steD* mutant or the complemented strain (Δ*steD* + p*steD*). As expected, the WT and Δ*steD* + p*steD* strains but not Δ*ssaV* or Δ*steD* mutants reduced MHCII surface levels in MutuDCs (Figs 2B and S1B). Similarly, WT and Δ*steD* + p*steD* strains reduced CD97 surface levels in comparison to cells infected with Δ*ssaV* or Δ*steD* mutants (Figs 2B and S1B).

Following translation, CD97 is autoproteolytically cleaved into an N-terminal, extracellular α subunit, which remains non-covalently linked to the C-terminal, transmembrane β subunit [15]. Available antibodies include a monoclonal recognising the CD97α subunit, suitable for flow cytometry, and polyclonal antibodies recognising the denatured CD97α or β subunits, which are suitable for immunoblotting. To determine the effect of SteD on these subunits, total levels of both were examined in infected MutuDCs by immunoblotting. Alternative splicing of CD97 mRNA generates at least three variants of the CD97α subunit (with expected masses of 58 kDa, 48 kDa and 44 kDa), all interacting with the single variant of the CD97β subunit [16]. Consistent with the flow cytometry experiments, infection of MutuDCs with WT or Δ*steD* + p*steD S*. Typhimurium caused a significant decrease in total CD97α and CD97β, whereas infection with the Δ*ssaV* and Δ*steD* mutants did not reproducibly affect the levels of either subunit (Fig 2C and 2D).

To examine if the effect of SteD on CD97 surface levels required MHCII, HEK293 cells (which do not express MHCII) were transfected with plasmids encoding CD97β-2HA (producing both CD97α and β subunits, with the intracellular C terminus of the β subunit tagged with a double HA epitope) and GFP or GFP-SteD. Quantification of surface CD97α in GFP^+^ cells by flow cytometry revealed that SteD decreased CD97 surface levels significantly; therefore its effect on CD97 is independent of MHCII (S1C Fig).

Next, the possible effect of SteD on CD97α was examined *in vivo* by flow cytometry. C57BL/6 mice were inoculated orally with GFP-expressing WT (WT-GFP) or Δ*steD* (Δ*steD*-GFP) *S*. Typhimurium and DCs were isolated from draining mesenteric lymph nodes (MLN) at 1, 3 or 6 days post-inoculation (p.i.). Quantification of surface CD97α revealed that DCs infected by the WT-GFP strain had significantly reduced levels of CD97α compared to DCs infected with the Δ*steD*-GFP strain at days 3 and 6 p.i. (Fig 2E) despite no detectable difference in bacterial load at either time point (S1D Fig). Collectively, these data indicate that CD97 is a physiological target of SteD.

### SteD interacts with CD97

**Fig 3:**
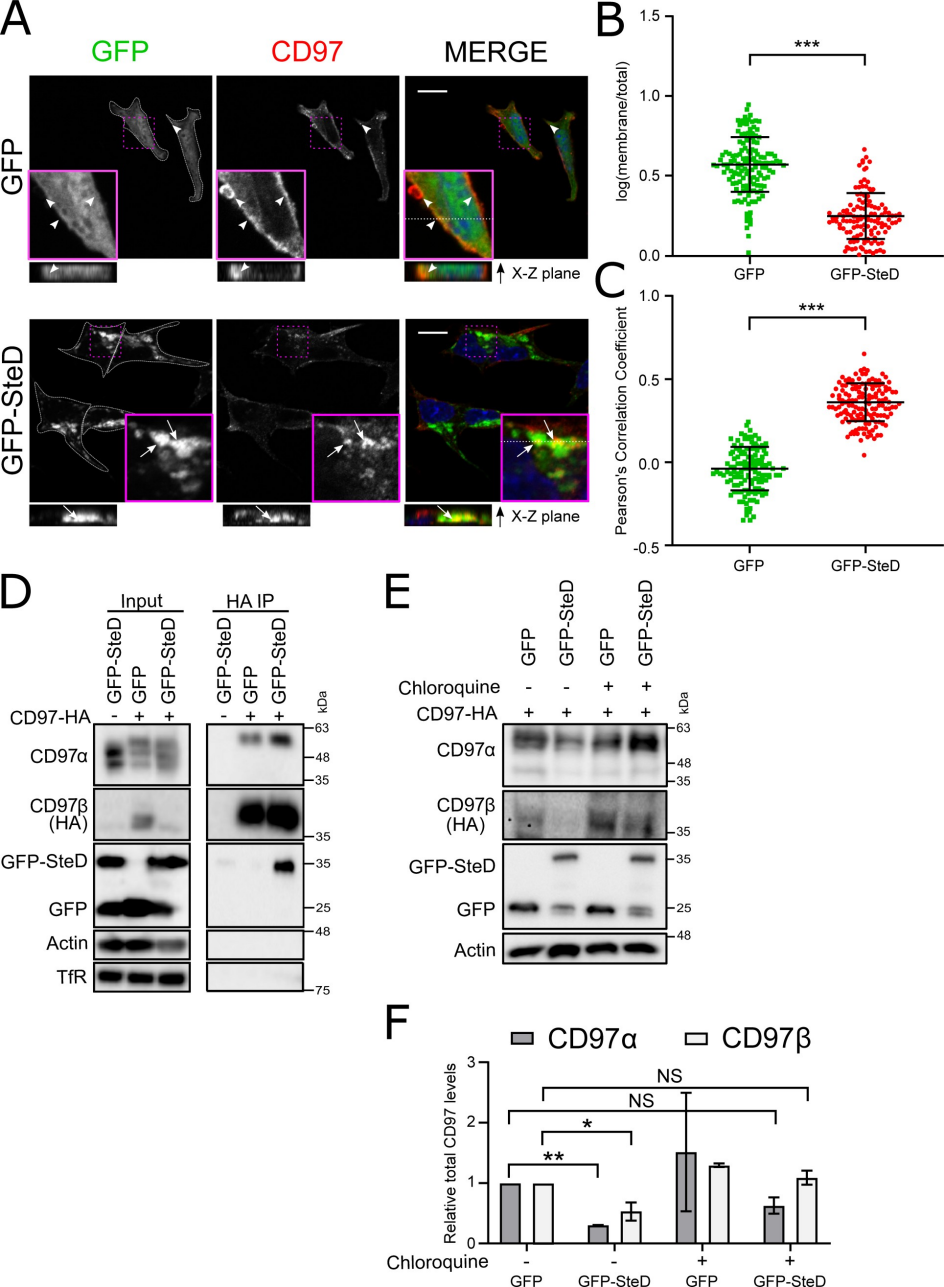
SteD forms a complex with CD97. (A) Representative confocal immunofluorescence microscopy images of total CD97α in CD97-2HA MutuDCs stably expressing either GFP or GFP-SteD (green) and activated with 100 ng/ml LPS. Cells were fixed 24 h post-activation, permeabilised with 0.01% TritonX-100 and labelled for CD97α (red) and DAPI (blue). Arrowheads indicate CD97α on plasma membrane and arrow indicates CD97α colocalising with GFP-SteD. 3D reconstruction from Z-stack images on the dotted line was used to identify intracellular signal. Scale bar—10 μm. (B) Ratio of membrane to total levels of CD97α in GFP or GFP-SteD expressing cells from 3 experiments represented in (A). Membrane signal was defined as a border of background cell fluorescence using CellProfiler software. Data are representative of three independent experiments. Each dot represents the value for one cell. Error bars show mean ± SD. *** p < 0.001 (Student’s T-test). (C) Quantification of colocalization between CD97-2HA and GFP in MutuDCs stably expressing CD97-2HA and either GFP or GFP-SteD. Cells were activated with 100 ng/ml LPS for 24 h. Error bars show mean ± SD. *** p < 0.001 (Student’s T-test). (D) Co-immunoprecipitation of GFP or GFP-SteD by anti-HA (CD97β -2HA) antibody. MutuDCs stably expressing CD97-2HA and either GFP or GFP-SteD were activated with 100 ng/ml LPS for 24 h prior to lysis and proteins were immunoprecipitated with anti-HA antibody. Samples were analysed by immunoblot using anti-CD97α, anti-HA (CD97β-2HA), anti-GFP, anti-transferrin receptor (TfR) and anti-actin antibodies. The slightly delayed migration of the co-immunoprecipitated CD97α is caused by different buffer composition (S2B Fig). (E) MutuDCs expressing CD97-2HA and either GFP or GFP-SteD, were mock-treated or exposed to 10 μM chloroquine for 24 h. Cell lysates were analysed by immunoblot using anti-CD97α, anti-HA (CD97β-2HA), anti-GFP and anti-actin antibodies. (F) Quantification of intensity of CD97α and β signal from 3 experiments represented in (E), normalised to that from GFP-expressing MutuDCs in absence of chloroquine. Error bars show means ± SD. * p < 0.05, ** p < 0.01, NS–not significant (two-way ANOVA followed by Tukey’s multiple comparison test).

To examine the localisation of CD97 and establish if SteD interacts with CD97, we constructed MutuDCs expressing CD97β-2HA. These were transduced to produce cells stably expressing GFP or GFP-SteD, then permeabilised and examined by immunofluorescence microscopy. In GFP-expressing cells, CD97 was mainly localised at the cell surface (Fig 3A, X-Z planes, and 3B), whereas in cells expressing GFP-SteD the CD97 labelling was predominantly intracellular (Fig 3A, X-Z planes, and 3B). Furthermore, the intracellular CD97 signal was punctate and frequently co-localised at its periphery with GFP-SteD (Fig 3A and 3C) suggesting that CD97α was within the lumen of multivesicular endosomes whose membranes contained SteD. These results show that SteD induces internalization and/or prevents recycling of CD97 and suggested that it might interact physically with CD97.

We then tested if SteD and CD97 interact by co-immunoprecipitation experiments. TfR, a transmembrane protein also present in CD97-containing vesicles (S2A Fig), but not affected by SteD [7], was not co-precipitated with CD97β-2HA by an anti-HA antibody (Fig 3D). GFP-SteD was not precipitated from MutuDCs not expressing CD97β-2HA, showing that GFP-SteD did not bind non-specifically to anti-HA agarose (Fig 3D), but was efficiently precipitated in the presence of CD97β-2HA (Fig 3D). The longest variant of CD97α (EGF1,2,X,3,4; [17] also interacted with CD97β-2HA (Fig 3D). Presumably this reflects the fact that the longest variant was overexpressed from the same construct as CD97β-2HA and lacks introns for alternative splicing and generation of shorter variants, endogenous forms of which were detected in cell lysates (Fig 3D). These results provide strong evidence that SteD forms a complex with CD97.

Plasma membrane protein turnover is often mediated by internalisation, ubiquitination, sorting into multivesicular bodies and lysosomal degradation [18–20]. To examine if SteD-mediated depletion of surface CD97 results in its lysosomal degradation, CD97-2HA MutuDCs either expressing GFP or GFP-SteD, were exposed to the lysosomal inhibitor chloroquine and CD97 protein levels were examined 24 h later by immunoblotting. In untreated cells, GFP-SteD caused a significant reduction in total CD97 levels (Fig 3E and 3F). This was rescued substantially by chloroquine treatment (Fig 3E and 3F) indicating that SteD induces lysosomal degradation of CD97.

### SteD requires Tmem127 and Wwp2 to deplete surface CD97

**Fig 4:**
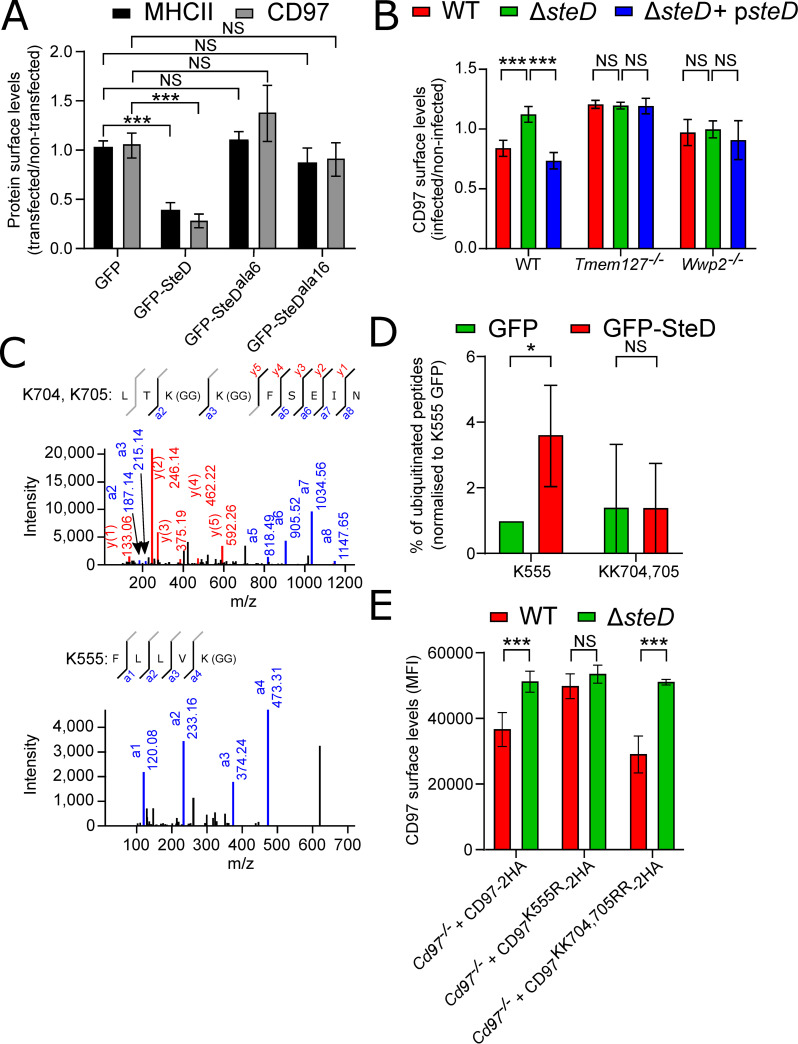
Tmem127 and Wwp2 are important for effect of SteD on CD97. (A) Quantification of MHCII and CD97α surface levels in MutuDCs expressing GFP, GFP-SteD, GFP-SteD^ala6^ or GFP-SteD^ala16^ and activated with 100 ng/ml LPS. Cells were analysed by flow cytometry 24 h post-activation and amounts of surface MHCII and CD97α in transfected cells are expressed as a fraction of fluorescence of non-transfected cells in the same sample. Data are from 3 independent experiments and show means ± SD. *** p < 0.001, NS—not significant (two-way ANOVA followed by Tukey’s multiple comparison test). (B) Quantification of CD97α surface levels in WT, *Tmem*127^-/-^ or *Wwp2*^-/-^ MutuDCs, infected with the indicated *S*. Typhimurium strains. Cells were analysed by flow cytometry 24 h p.i. and amounts of surface CD97α in infected cells are expressed as a fraction of fluorescence of non-infected cells in the same sample. Data are from 3 independent experiments and show means ± SD. *** p < 0.001, NS—not significant (two-way ANOVA followed by Tukey’s multiple comparison test). (C) MALDI MS/MS spectrum of peptides obtained after trypsinization of endogenous CD97 obtained from MutuDCs cells stably expressing GFP-SteD. K704, K705 and K555 are modified with a G-G branch [K(GG)] indicating ubiquitination at these positions. (D) Percentage of peptides analysed in (C) modified with a G-G branch [K(GG)] indicating ubiquitination at these positions normalised to percentage of modified K555 peptides in the GFP-expressing sample. * p < 0.05, NS–not significant (Student’s T-test). (E) Quantification of CD97α surface levels in *Cd97*^*-/-*^ + CD97-2HA, *Cd97*^*-/-*^ + CD97^K555R^-2HA or *Cd97*^*-/-*^ + CD97^KK704,705RR^-2HA MutuDCs, infected with the indicated *S*. Typhimurium strains. Cells were analysed by flow cytometry 24 h p.i. and amounts of surface CD97α in infected cells are expressed as median fluorescence intensity (MFI). Data are from 3 independent experiments and show means ± SD. *** p < 0.001, NS—not significant (two-way ANOVA followed by Tukey’s multiple comparison test).

To examine the mechanism by which SteD induces CD97 lysosomal degradation, we stably transduced MutuDCs with two *steD* mutants, whose products (GFP-SteD^ala6^ and GFP-SteD^ala16^) are unable to induce MHCII degradation in Mel JuSo cells [7]. As expected, neither GFP-SteD^ala6^ nor GFP-SteD^ala16^ reduced MHCII surface levels in MutuDCs (Fig 4A). Similarly, these mutants were defective in reducing CD97α surface levels (Fig 4A). While the function of the SteD ala6 region remains uncharacterised, the ala16 region is required for interactions with the transmembrane protein Tmem127 [8]. Tmem127 is a Nedd4 family interacting protein (NDFIP) for the Nedd4 family HECT E3 ubiquitin ligase Wwp2. Therefore, we examined the requirement of Tmem127 and Wwp2 for the effect of SteD on CD97. Neither loss of Tmem127 nor Wwp2 affected surface levels of CD97α in LPS-activated MutuDCs (S3A Fig). However, the absence of Tmem127 or Wwp2 prevented the SteD-dependent decrease of CD97α surface levels (Figs 4B and S3B).

### SteD induces ubiquitination of CD97

The requirement for Tmem127 and Wwp2 suggested that SteD recruits these proteins to induce ubiquitination of CD97, as occurs for mMHCII [8]. Analysis of mass spectrometry data on CD97 revealed that in the presence or absence of SteD, three residues in the CD97β subunit (K555, K704, K705) contained di-glycyl remnants, indicating that they were ubiquitinated (Fig 4C). The presence of SteD did not lead to reproducible ubiquitination of CD97 on any other detected residue. The proportion of CD97 peptides containing K704 and K705 with di-glycyl modifications was not influenced by the presence of SteD. However, the proportion of CD97 peptides that contained di-glycyl K555 was increased in the presence of SteD (Fig 4D).

To investigate the functional significance of K555-ub and K704, K705-ub, we constructed *Cd97*^-/-^ MutuDCs (S3C Fig) and used these for stable expression of wild-type CD97-2HA, CD97^K555R^-2HA or CD97^KK704,705RR^-2HA mutants (S3D Fig). There was no significant difference between CD97α surface levels of the three overexpressed CD97 variants (S3E Fig). As expected, the CD97α surface level of wild-type *S*. Typhimurium-infected *Cd97*^-/-^ + CD97-2HA cells was lower when compared to the same cells infected with Δ*steD S*. Typhimurium (Fig 4E). Similarly, wild-type but not Δ*steD S*. Typhimurium decreased the CD97α surface levels in *Cd97*^-/-^ + CD97^KK704,705RR^-2HA MutuDCs. However, SteD did not affect the CD97α surface levels in *Cd97*^-/-^ + CD97^K555R^-2HA MutuDCs (Fig 4E), indicating that K555 is critical for ubiquitination.

### SteD inhibits activation of T cells

To gain further insights into possible functional consequences of the effect of SteD on CD97, we characterised surface levels of CD97α in DC subpopulations in draining MLN of C57BL/6 mice orally inoculated with WT-GFP or Δ*steD*-GFP *S*. Typhimurium. In draining MLNs, expression of CD103 and CD11b surface markers define three major DC subpopulations (CD103^+^CD11b^-^, CD103^+^CD11b^+^, CD103^-^CD11b^+^), which differ in their capacity to activate specific T cell subsets [21], [22]. All three DC subpopulations were infected to similar degrees 6 days p.i., and CD97α surface levels were reduced by SteD in each subpopulation (S4A Fig).

**Fig 5:**
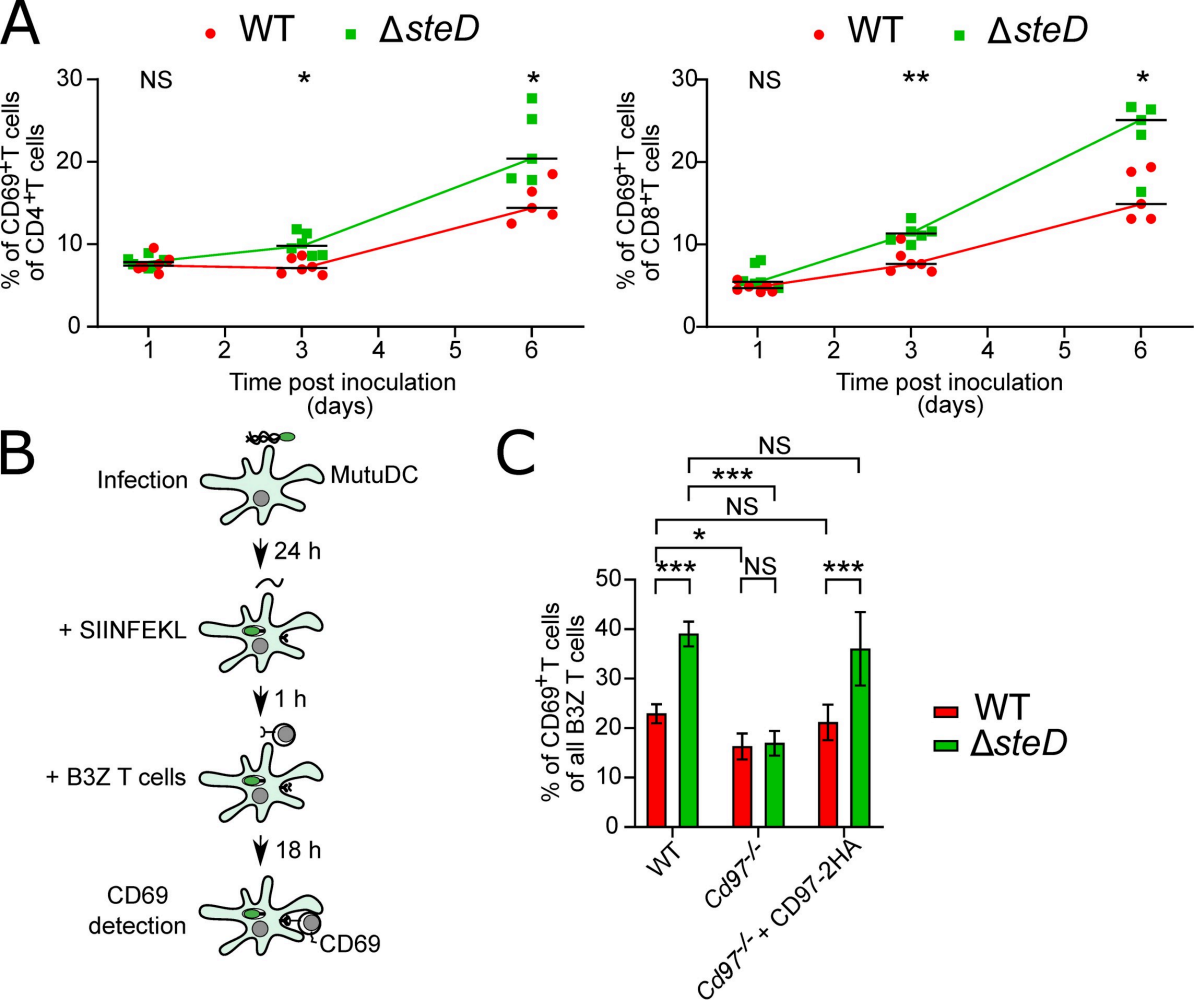
SteD interferes with CD97-dependent activation of T cells. (A) Quantification of CD69^+^CD4^+^ and CD69^+^CD8^+^ T cells in infected mice. Cells were isolated from draining MLNs of C57BL/6 mice inoculated orally with the indicated *S*. Typhimurium strains at indicated times post-inoculation. Amounts of CD69^+^ T cells are expressed as percentage of all CD11c^-^CD3^+^CD4^+^ or CD11c^-^CD3^+^CD8^+^ cells. Each dot represents the value obtained from MLNs pooled from two or three mice from three independent experiments and means ± SD are shown. * p < 0.05, ** p < 0.01, NS—not significant (two-way ANOVA followed by Tukey’s multiple comparison test). (B) Schematic of *in vitro* SIINFEKL (OVA)-dependent B3Z T cell activation assay. MutuDCs infected with GFP-expressing *S*. Typhimurium were exposed to SIINFEKL (ovalbumin-derived peptide for MHCI presentation). After 1 h, CD8^+^ B3Z T cells were added and co-incubated for 18 h prior to measurement of CD69 on B3Z T cells by flow cytometry. (C) *In vitro* SIINFEKL (OVA)-dependent B3Z T cell activation assay. WT, *Cd97*^*-/-*^ or *Cd97*^*-/-*^ + CD97-2HA MutuDCs were infected with WT-GFP or Δ*steD*-GFP *S*. Typhimurium and exposed to SIINFEKL peptide. Percentage of CD69^+^ B3Z T cells in each condition is shown. Data are from 3 independent experiments and show means ± SD. * p < 0.05, *** p < 0.001, NS–not significant (two-way ANOVA followed by Tukey’s multiple comparison test).

Direct stimulation of CD55 on CD4^+^ T cells leads to expression of the T cell activation marker CD69. This is thought to be driven by interaction of CD55 with CD97 [23]. To examine the effects of SteD on CD69 surface levels on T cells *in vivo*, C57BL/6 mice were inoculated orally with WT-GFP or Δ*steD*-GFP *S*. Typhimurium and T cells were isolated from mesenteric lymph nodes at 1, 3 or 6 days p.i. At these time-points, SteD did not influence bacterial loads in MLN or spleens (S1D Fig). Similarly, at 6 days p.i., there was no influence of SteD on CD4^+^/CD8^+^ ratios in *S*. Typhimurium*-*colonised MLNs (S4B Fig). However, both CD4^+^ and CD8^+^ T cells isolated from Δ*steD*-GFP-inoculated mice had significantly higher levels of surface CD69 compared to CD4^+^ and CD8^+^ T cells isolated from WT-GFP inoculated mice (Figs 5A and S4C). The reduction of CD69 surface levels in both CD4^+^ and CD8^+^ T cells suggested that the effect of SteD is independent of its effect on MHCII but might be related to an effect on MHCI. To test this, MutuDCs stably expressing GFP or GFP-SteD or infected with WT-GFP or Δ*steD*-GFP *S*. Typhimurium for 24 h were incubated with the MHCI specific OVA peptide SIINFEKL. Flow cytometry using an antibody specifically recognising SIINFEKL-loaded MHCI showed that SteD did not influence OVA presentation by MHCI (S5A Fig).

Because MHCI antigen presentation was not affected by SteD, we used an MHCI-specific antigen presentation assay to analyse the effect of SteD on T cell CD69 surface levels independently of its known effect on MHCII antigen presentation. To do this, we incubated B3Z T cells, a commonly used CD8^+^ T cell line, with SIINFEKL-loaded MutuDCs for 18 hours and then measured levels of CD69 on the surface of B3Z T cells (Fig 5B). B3Z T cells co-incubated with SIINFEKL-loaded and WT-GFP *S*. Typhimurium-infected MutuDCs had significantly less surface CD69 than B3Z T cells co-incubated with SIINFEKL-loaded MutuDCs containing Δ*steD*-GFP *S*. Typhimurium (Figs 5C and S5B). This shows that the *in vitro* MutuDC-B3Z T cell interaction model recapitulates results obtained *in vivo* and that the effect of SteD on CD8^+^ T cell CD69 is not mediated through CD4^+^ T cells. To address the hypothesis that decreased T cell surface levels of CD69 in response to DCs containing WT-GFP *S*. Typhimurium is dependent on CD97, *Cd97*^-/-^ MutuDCs and *Cd97*^*-/-*^ + CD97-2HA MutuDCs were used. SIINFEKL-loaded *Cd97*^-/-^ MutuDCs induced significantly lower surface levels of CD69 in B3Z T cells regardless of the *S*. Typhimurium strain (Figs 5C and S5B). In contrast, Δ*steD*-GFP *S*. Typhimurium infection of both WT and *Cd97*^-/-^ + CD97-2HA MutuDCs induced significantly higher surface levels of CD69 from B3Z T cells, compared to T cells exposed to WT-GFP *S*. Typhimurium-infected WT and *Cd97*^-/-^ + CD97-2HA MutuDCs (Figs 5C and S5B). These results show that the SteD-mediated decrease in surface levels of CD97α is responsible for decreased CD69 surface levels on T cells, independently of the effect of SteD on MHCII.

### SteD reduces DC-T cell interactions

**Fig 6:**
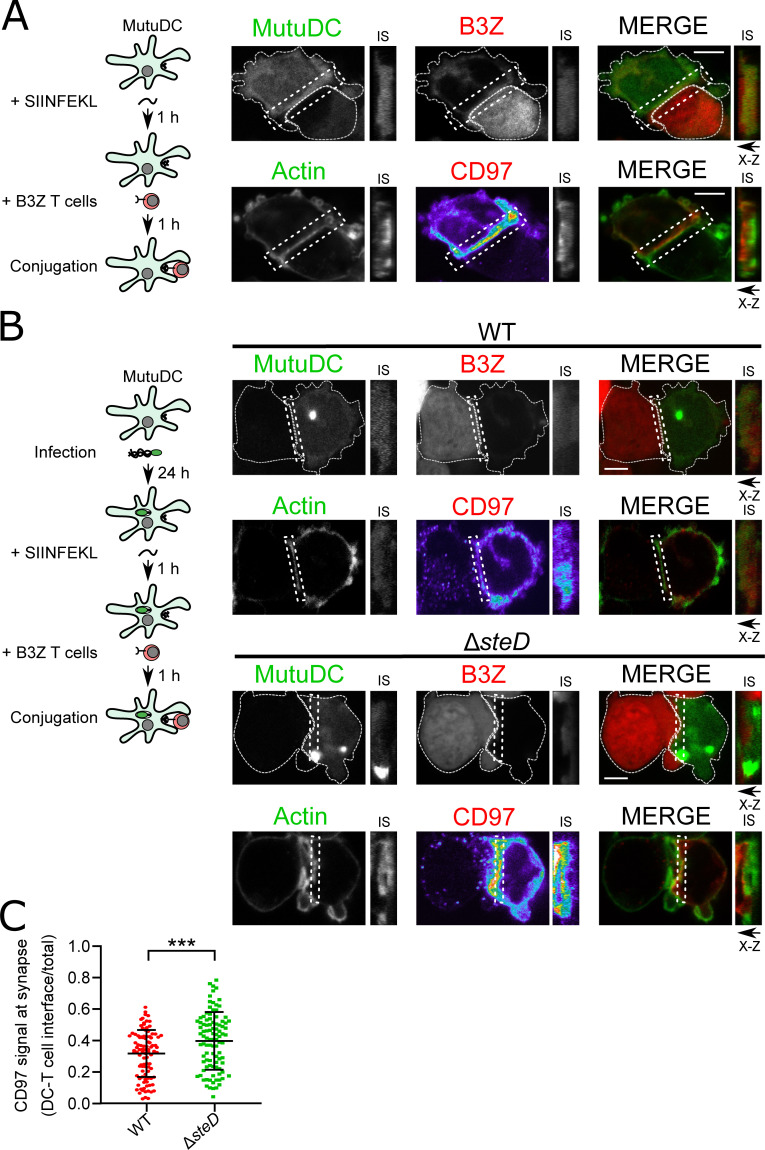
SteD reduces levels of CD97 at the IS. (A) Left: Schematic of *in vitro* SIINFEKL (OVA)-dependent B3Z T cell conjugation assay for microscopy. *Cd97*^*-/-*^ + CD97-2HA MutuDCs were exposed to SIINFEKL for 1 h before co-incubation with B3Z T cells. After 1 h, DC-T cell conjugates were fixed and analysed by confocal microscopy. (B) Right: Representative confocal immunofluorescence microscopy images of CD97β-2HA localisation at the IS. *Cd97*^*-/-*^ + CD97-2HA MutuDCs (GFP, green, top panels) were incubated with SIINFEKL then exposed to CellTracker Blue-labelled B3Z T cells (red, top panels) for 1 h. Anti-HA antibody was used to detect CD97β-2HA (heat map in the single colour channel illustrates the gradient of accumulation of CD97β-2HA signal at the IS; red in the merge, bottom panels). Phalloidin labelling of F-actin (green, bottom panels) was used to define the synaptic region (IS). X-Z projections (at the side of the main images) were used to show the IS. Scale bar—5 μm. (C) Left: Schematic of *in vitro* SIINFEKL (OVA)-dependent B3Z T cell conjugation assay on infected MutuDCs for microscopy. *Cd97*^*-/-*^ + CD97-2HA MutuDCs infected with GFP-expressing *S*. Typhimurium were exposed to SIINFEKL for 1 h before co-incubation with B3Z T cells. After 1 h, DC-T cell conjugates were fixed and analysed by confocal microscopy. Right: Representative confocal immunofluorescence microscopy images of CD97β-2HA localisation at the IS following infection. *Cd97*^*-/-*^ + CD97-2HA MutuDCs (GFP, green, top panels) infected with WT-GFP or Δ*steD*-GFP *S*. Typhimurium were incubated with SIINFEKL then exposed to CellTracker Blue-labelled B3Z T cells (red, top panels) for 1 h. Anti-HA antibody was used to detect CD97β-2HA (heat map in the single colour channel illustrates the gradient of accumulation of CD97β-2HA signal at the IS; red in the merge, bottom panels). Bright GFP puncta represent bacteria oriented in the Z axis (S6C Fig). Phalloidin labelling of actin (green, bottom panels) was used to visualise IS. X-Z projection was used to show the IS. Scale bar—5 μm. (C) Quantification of CD97β-2HA at the IS from 3 experiments represented in (B). The synaptic region was identified by F-actin at the DC-T cell interface and the ratio of CD97β-2HA signal at the DC-T cell interface to the total CD97β-2HA signal was calculated using CellProfiler software. Each dot represents the value for one cell. Error bars show mean ± SD. *** p < 0.001 (Student’s T-test).

CD97 interacts with extracellular matrix and mediates cell-cell interactions by binding to complement decay-accelerating factor DAF/CD55 [24]. To examine the localisation of CD97 during DC-T cell interactions, SIINFEKL-loaded *Cd97*^-/-^ + CD97-2HA MutuDCs were co-incubated with CellTracker Blue-loaded B3Z T cells and examined by confocal immunofluorescence microscopy (Fig 6A left panel). The accumulation of F-actin at the cell-cell interface helped define the IS (Figs 6A, right panel, and S6A). This revealed a very strong recruitment of CD97β-2HA to the synaptic region. In the synaptic region, CD97β-2HA did not co-localise with actin filaments, presumably because the latter are cytoplasmic assemblies while CD97 is located within the plasma membrane (Figs 6A and S6A).

**Fig 7:**
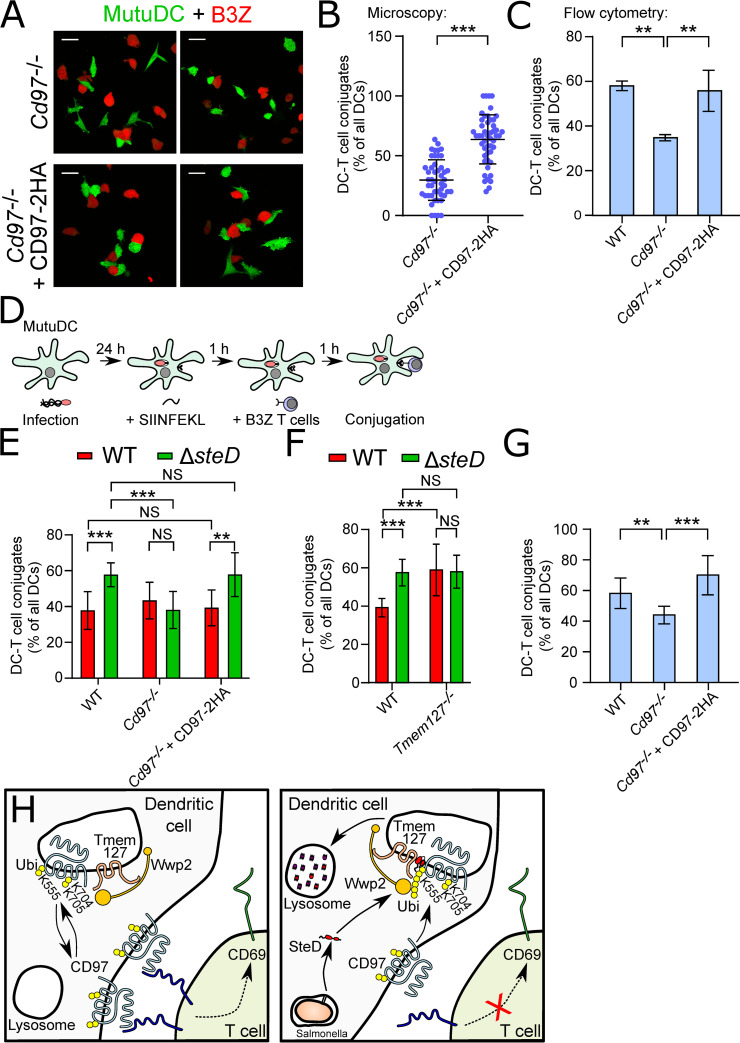
SteD compromises CD97-dependent stabilisation of the DC-T cell interaction. (A) Representative confocal immunofluorescence microscopy images of DC-T cell conjugation by confocal microscopy. *Cd97*^*-/-*^ or *Cd97*^*-/-*^ + CD97-2HA MutuDCs (GFP^+^) exposed to SIINFEKL peptide were allowed to interact with CellTracker Blue-labelled B3Z T cells (falsely coloured red) for 1 h. Two representative examples for each MutuDC variant are shown. Scale bar—20 μm. (B) Quantification of DC-T cell conjugation from 3 experiments represented in (A). The DC-T cell conjugates were identified based on MutuDC-inherent GFP signal and B3Z CellTracker Blue signal. Percentage of DC-T cell conjugates per all MutuDCs was calculated in 50 fields of view (at magnification 63x) containing around 500 MutuDCs. Each dot represents one field of view. Error bars show mean ± SD. *** p < 0.001 (Student’s T-test). (C) *In vitro* SIINFEKL (OVA)-dependent MutuDC-B3Z T cell conjugation assay. WT, *Cd97*^*-/-*^ or *Cd97*^*-/-*^ + CD97-2HA MutuDCs (GFP^+^) exposed to SIINFEKL peptide were allowed to interact with CellTracker Blue-labelled B3Z T cells. Percentage of GFP^+^ (MutuDCs) and CellTracker Blue^+^ (B3Z) double positive conjugates as a fraction of all GFP^+^ events for each condition representing is shown. Data are from 3 independent experiments and show means ± SD. ** p < 0.01 (one-way ANOVA followed by Tukey’s multiple comparison test). (D) Schematic of *in vitro* SIINFEKL (OVA)-dependent MutuDC-B3Z T cell conjugation assay on infected MutuDCs for flow cytometry. *S*. Typhimurium strains constitutively expressing mCherry from pFCcGi were used to distinguish non-infected MutuDCs (inherently GFP^+^) and infected MutuDCs (mCherry^+^, GFP^+^). (E) SIINFEKL (OVA)-dependent conjugation assay between infected MutuDCs and B3Z T cells conjugation assay *in vitro*. WT, *Cd97*^*-/-*^ or *Cd97*^*-/-*^ + CD97-2HA MutuDCs (GFP^+^) were infected with WT-mCherry or Δ*steD*-mCherry *S*. Typhimurium and incubated with SIINFEKL peptide, then exposed to CellTracker Blue-labelled B3Z T cells. The percentages of infected MutuDCs (mCherry^+^GFP^+^) and B3Z (CellTracker Blue^+^) triple positive conjugates are shown as a fraction of all infected MutuDCs (mCherry^+^GFP^+^ events) for each condition. Data are from 3 independent experiments and show means ± SD. ** p < 0.01, *** p < 0.001, NS–not significant (two-way ANOVA followed by Tukey’s multiple comparison test). (F) SIINFEKL (OVA)-dependent MutuDC-B3Z T cells conjugation assay *in vitro*. WT or *Tmem127*^*-/-*^ MutuDCs (GFP^+^) infected with WT-mCherry or Δ*steD*-mCherry *S*. Typhimurium and incubated with SIINFEKL peptide were exposed to CellTracker Blue-labelled B3Z T cells. Percentage of infected MutuDCs (mCherry^+^GFP^+^) and B3Z (CellTracker Blue^+^) triple positive conjugates are shown as a fraction of all infected MutuDCs (mCherry^+^GFP^+^ events) for each condition. Data are from 3 independent experiments and show means ± SD. *** p < 0.001, NS–not significant (two-way ANOVA followed by Tukey’s multiple comparison test). (G) ISQAVHAAHAEINEAGR (OVA)-dependent MutuDC-CD4^+^ T cell conjugation assay *in vitro*. WT, *Cd97*^*-/-*^ or *Cd97*^*-/-*^ + CD97-2HA MutuDCs (GFP^+^) incubated with ISQAVHAAHAEINEAGR peptide were exposed to primary CellTracker Blue-labelled CD4^+^ T cells expressing OVA-specific T cell receptor. GFP^+^ (MutuDCs) and CellTracker Blue^+^ (CD4^+^ T cells) double positive conjugates are shown as a percentage of all GFP^+^ events. Data are from 3 independent experiments and show means ± SD. ** p < 0.01 (one-way ANOVA followed by Tukey’s multiple comparison test). (H) Model for mechanism of CD97 surface depletion by SteD. Left: Tmem127 (beige) interacts with the E3 ubiquitin ligase Wwp2 (orange). In the absence of SteD, CD97 (light blue) recycles to the plasma membrane, where it interacts with a T cell surface protein (blue), stabilising IS and triggering expression of the activation marker CD69 (green). Right: The presence of SteD (red) stimulates Wwp2-dependent ubiquitination (yellow) of CD97 and its subsequent lysosomal degradation, destabilising the DC-T cell interaction.

To determine if SteD causes removal of CD97 from the IS, *Cd97*^-/-^ + CD97-2HA MutuDCs infected with WT-GFP or Δ*steD*-GFP *S*. Typhimurium and exposed to SIINFEKL were co-incubated with B3Z T cells (Fig 6B left panel). Confocal immunofluorescence microscopy of these cells showed that while F-actin accumulation was unaffected by SteD (S6B Fig), there was a significantly lower proportion of CD97β-2HA at the synapses of *Cd97*^-/-^ + CD97-2HA MutuDCs containing WT-GFP *S*. Typhimurium compared to synapses involving the Δ*steD*-GFP infected DCs and B3Z T cells (Fig 6B, right panel, and 6C). To determine if removal of CD97 from the IS affected DC-T cell interactions, we quantified the amount of conjugation between MutuDCs and B3Z T cells after SIINFEKL-stimulation in the absence of CD97 by both microscopy and flow cytometry. CD97 knock-out cells were defective in the formation or stability of MutuDC-B3Z T cell conjugates, and restoration of CD97 expression rescued this defect (Fig 7A-7C).

To test if SteD-mediated removal of CD97 from the cell surface was sufficient to affect the physical interaction between DCs and T cells, WT, *Cd97*^-/-^ or *Cd97*^-/-^ + CD97-2HA MutuDCs were infected with *S*. Typhimurium strains for 24 h, exposed to SIINFEKL, then co-incubated with B3Z T cells and analysed as above (Fig 7D). Infection of WT and *Cd97*^*-/-*^ + CD97-2HA MutuDCs with WT-mCherry *S*. Typhimurium decreased significantly the amount of MutuDC-B3Z T cell conjugates compared to Δ*steD*-mCherry *S*. Typhimurium, whose effect was similar to cells that had not been exposed to bacteria (Fig 7C and 7E). In contrast, *Cd97*^-/-^ MutuDCs interacted less with B3Z T cells regardless of whether they contained WT-mCherry or Δ*steD*-mCherry *S*. Typhimurium (Figs 7E and S7A). Since the effect of SteD on CD97 is dependent on Tmem127, we examined the influence of *S*. Typhimurium infection on conjugate formation in *Tmem127*^-/-^ MutuDCs. Deletion of *Tmem127* did not affect MutuDC-B3Z T cell conjugation in the absence of *Salmonella* (S7B Fig). However, the ability of WT-mCherry *S*. Typhimurium to decrease DC-T cell conjugate formation was lost in the presence of *Tmem127*^-/-^ MutuDCs (Fig 7F). These results indicate that the SteD- and Tmem127-dependent decrease of CD97 surface levels in DCs weakens their interaction with CD8^+^ T cells.

To test the involvement of CD97 in stabilizing DC-T cell interactions involving MHCII, we quantified conjugation between WT, *Cd97*^-/-^ or *Cd97*^-/-^ + CD97-2HA MutuDCs exposed to the MHCII-specific OVA peptide ISQAVHAAHAEINEAGR and OVA-specific CD4^+^ T cells isolated from spleens of OTII mice. CD97 knock-out cells were defective in the formation or stability of MutuDC-CD4^+^ T cell conjugates, and restoration of CD97 expression rescued this defect (Fig 7G) confirming that CD97-dependent formation or stabilisation of the IS, is independent of antigen presentation.

Collectively, our results have revealed that in dendritic cells, CD97 localises to and stabilises the IS. SteD interferes with this by inducing the removal of surface CD97 through its ubiquitination and degradation by the Tmem127/Wwp2 machinery, thereby reducing DC-T cell interactions and inhibiting T cell activation independently of its effect on MHCII (Fig 7H).

## Discussion

In this work we carried out a systematic, unbiased proteomic screen for additional cell surface targets of SteD. We found that when SteD was overexpressed as a GFP-fusion protein, in addition to both α and β chains of the H2-A isoform of MHCII, CD97 and Il1rap levels were reduced. The physiological relevance of CD97 depletion was validated in MutuDCs infected with *Salmonella* mutant strains. Since Il-1β signalling is important for control of *Salmonella* infection in the intestine [25,26] and Il1rap is necessary for formation of a functional Il1 receptor, we intend to assess the physiological effect of SteD on Il1rap in future studies.

Our evidence indicates that SteD recruits the adaptor protein Tmem127 and its cognate E3 ubiquitin ligase Wwp2 to induce ubiquitination of a cytoplasmic lysine residue of CD97β, leading to its lysosomal degradation. Thus, SteD depletes surface CD97 by a mechanism similar to that used on mMHCII. There is no obvious amino acid similarity in the regions immediately flanking the residues in these two substrates whose ubiquitination was enhanced by SteD: K555 of CD97 and K225 of the beta chain of MHCII. However, while CD97 has seven transmembrane domains, K555 (and the two other ubiquitinated residues K704, K705) are all exposed to the host cell cytoplasm and are therefore potentially accessible to host cell ubiquitination machinery. The identification of three different cytoplasmic lysine residues in the CD97β subunit that undergo ubiquitination in the presence or absence of SteD strongly suggests that turnover of the protein is regulated by ubiquitination.

Three ligands (α5β1 integrin, chondroitin sulphate and CD55) have been identified for human CD97, the best characterised of which is CD55 [24,27,28]. In contrast to human CD97, mouse CD97 lacks the RGD motif necessary for integrin binding and its ability to bind chondroitin sulphate has yet to be established [16]. CD55 is therefore the only validated ligand of mouse CD97 [16]. Antibodies to both CD55 and CD97 inhibit T cell proliferation and IFN-γ secretion [29]. Stimulation of CD55 on CD4^+^ T cells leads to expression of the T cell activation marker CD69 [23]. To assess the effects of SteD-dependent depletion of CD97 on CD69, it was necessary to design the experiment so that the potentially confounding influence of SteD on MHCII was prevented. To do so we took advantage of an MHC class I-CD8 T cell specific assay, having shown that SteD does not affect peptide-loaded MHC class I surface levels, in agreement with previous work suggesting that *Salmonella* does not target this molecule [30]. We then examined the effects of depletion of CD97 from dendritic cells on surface levels of CD69 in T cells. This revealed that SteD reduced CD69 surface levels in a CD97-dependent manner. Furthermore, surface levels of CD69 in CD4^+^ and CD8^+^ T cells were significantly lower in MLNs of mice infected with wild-type *Salmonella* compared to the *steD* mutant, indicating that this effect also occurs during natural infection. While CD4^+^ T cells represent a key component of adaptive immunity against *Salmonella*, it has been shown recently that CD8^+^ T cells can also confer protection, at least in certain mouse strains [4]. Therefore, it is possible that in addition to its effect on CD4^+^ T cells, depletion of CD69 from CD8^+^ T cells is relevant to *Salmonella* virulence. Although robust initial activation of both CD8^+^ and CD4^+^ T cells occurs initially in response to *Salmonella* infection [31], we previously detected significant SteD-mediated suppression of CD4^+^ T cell activity at day 17 days post-oral inoculation [7]. Interestingly, others have detected unexpectedly low levels of T cell proliferation in response to *Salmonella* infection *in vivo*, despite these cells having an activated phenotype [32]. In agreement with this, we found that SteD decreased proliferation of antigen-specific CD4^+^ T cells *in vitro* [7,8]. The *in vitro* experiments reported here, showing that the effect on CD69 is independent of MHC-TCR interactions agrees with the results of our co-immunoprecipitation experiments, which revealed a physical interaction between SteD and CD97.

In contrast to *in vitro* studies, the contribution of CD97 to T cell responses *in vivo* are less clear. Lung infection of *Cd97* knock-out mice by *Streptococcus pneumoniae* does not result in a difference in short-term growth, compared to wild-type mice [33]. Another study showed that *Cd97* knock-out mice are more resistant to infection by *Listeria monocytogenes* at early stages, and this was attributed in part to a small increase in blood granulocytes, which is a feature of these mice [34]. In a limited assessment of mouse survival at 3 months post-challenge, lack of *Cd97* did not seem to affect adaptive immunity [34]. However, it is not possible to extrapolate from these studies on Gram-positive bacteria to *Salmonella*, which causes a very different systemic disease in mice, characterised by survival and replication within macrophages [35]. The basal granulocytosis phenotype also complicates analysis of the contribution of *Cd97* in adaptive immunity to bacterial infection, but development of *Cd97* conditional knock-out mice could help circumvent this problem.

A major finding from this work is that CD97 localises to and helps form and/or stabilize the IS between DCs and T cells. The IS is a complex structure composed of spatially segregated substructures whose formation and maintenance is dependent on several intercellular protein/protein interactions, including CD80-CD28 and particularly ICAM1-LFA-1 at the DC/T cell interface [36–38]. The actin cytoskeleton also has a key role in its formation [39], and its perturbation in T cells by *Shigella* affects their ability to scan B cells efficiently, which reduces cell-cell conjugation [40]. Despite the existence of *Salmonella* effectors that target actin [1], we have not observed an obvious effect of *Salmonella* on F-actin accumulation in the synaptic region of DCs. In view of the numerous and complex protein-protein interactions at the IS, it is remarkable that deletion of CD97 alone results in a significant defect in DC-T cell conjugation (Fig 7A–7C). More work will be needed to determine the sub-structural localisation of CD97 at the synapse, if as previously suggested [29], the relevant interaction partner for CD97 is CD55, and/or if integrin binding is involved.

In conclusion, a proteomic screen revealed that in addition to MHCII and CD86, SteD has a third target: CD97. It is striking that SteD interferes with T cell activation by targeting two host membrane proteins that have independent functions at the IS (antigen presentation and synapse stabilisation), and this highlights the level of mechanistic sophistication by which *Salmonella* suppresses adaptive immune responses. The cumulative effects of SteD are likely to confer a strong selective advantage to *Salmonella* during long-term and chronic systemic infections and the high degree of functional conservation of *steD* among serovars of this pathogen [1] is likely to reflect this.

## Materials and methods

### Ethics statement

Experiments involving infection of C57BL/6 mice were conducted in accordance with European Directive 2010/63/EU regulations with approval from Imperial College London, Animal Welfare and Ethical Review Body (ICL AWERB) under the Personal Project license of David Holden.

### Bacterial strains

Bacteria were grown in Luria–Bertani (LB) medium supplemented with carbenicillin (50 μg/ml), kanamycin (50 μg/ml) or chloramphenicol (30 μg/ml) as appropriate. See key resources table for all bacterial strains.

### Cell culture

MutuDCs [10], which express GFP weakly, under the control of CD11c promoter, and their variants were maintained in IMDM-glutamax (GIBCO 31980), supplemented with 8–10% heat inactivated, endotoxin-free FCS, 10 mM HEPES pH 7.4 and 50 μM β-mercaptoethanol.

B3Z T cells expressing OVA-specific T cell receptor [41] were maintained in RPMI1640 supplemented with 8–10% heat inactivated, endotoxin-free FCS, 10 mM HEPES pH 7.4, sodium pyruvate and 50 μM β-mercaptoethanol.

### Cell infection

For infection of MutuDCs, overnight Luria broth (LB) cultures of *S*. Typhimurium strains (OD_600_ ~ 2.5 to 3.0) were added to cells at an MOI of 20:1, centrifuged at 110 *g* for 5 min and incubated at 37°C for 30 min. Cells were washed 2 times with PBS and incubated in fresh medium containing gentamicin (100 μg/ml) for 1 h to kill extracellular bacteria. After 1 h, the antibiotic concentration was reduced to 20 μg/ml, and cells were processed 24 h post-infection (p.i.).

### Mice

6 to 8 week old female C57BL/6 and OT-II mice (Charles River) were housed as 5 mice per individually ventilated cage under Specified Pathogen Free conditions. For infection experiments, mice were inoculated by oral gavage with 1x10^10^ colony forming units (CFU) of stationary phase *S*. Typhimurium 12023 + pGcDi (either WT or Δ*steD*::Km; OD_600_ ~ 2.5 to 3.0) in 200 μl of PBS containing 3% NaHCO_3_ or with 200 μL of sterile PBS containing 3% NaHCO_3_. Mesenteric lymph nodes were isolated at indicated times after inoculation and enzymatically dissociated in the presence of collagenase IV (1 mg/ml) and DNAse I (0.1 mg/ml) in HBSS supplemented with 10 mM HEPES and 2% FCS for 30 min at 37°C. Enzymatic digestion was stopped with addition of EDTA (2 mM final concentration). Cells were subsequently collected through a 70 μm cell strainer on ice and washed twice with Ca^2+^, Mg^2+^ free HBSS supplemented with 10 mM HEPES and 2% FCS and used for flow cytometry analysis.

### Biotinylation of cell surface proteins

For Aminooxy-Biotinylation, MutuDCs (4 x 10^7^) stably expressing either GFP or GFP-SteD were activated with 100 ng/ml of LPS 24 h prior to harvesting from cell culture plates using 2 mM EDTA in PBS. After gentle washing, the cells were resuspended in ice cold biotinylation mix (1 mM Sodium periodate, 5 ng/ml aminooxy-biotin, 0.1% aniline in PBS pH 6.7) and incubated with gentle rocking for 30 min at 4°C to restrict aminooxy-biotin uptake. The biotinylation reaction was then stopped with addition of 10 mM glycerol. Cells were washed carefully 3 times with ice cold Tris pH 7.4 and stored at -80°C. Pellets were resuspended in 1 ml cold lysis buffer (10 mM Tris pH 8.0, 150 mM NaCl, 1% Triton X-100) containing protease inhibitor cocktail (Roche Complete, EDTA free) and incubated with end-over-end rotation at 4°C for 30 min. Nuclei were removed by centrifuging twice at 13,000 *g* for 10 min at 4°C. Supernatants were added to 50 μl of High Capacity Streptavidin-Agarose beads (Thermo Fisher) and incubated at 4°C for 2 h with end-over-end rotation. Beads were then transferred to 500 μL Snap-Cap filter units (Thermo Fisher) and placed on a vacuum manifold for washing. Beads were washed with 400 μl volumes of the following buffers: 20x with lysis buffer, 20x with 50mM Tris 8.0, 0.5% SDS, 10x with 50 mM TEAB, 6M urea. Beads were then resuspended in urea wash buffer containing 10 mM TCEP and 40 mM Iodoacetamide and incubated with agitation at room temperature for 45 min. Subsequently a further 10x washes with urea buffer and 3x washes with 50 mM TEAB were made. Washed beads were transferred to clean tubes and resuspended in 50 μl 50 mM TEAB containing 0.5 μg trypsin (proteomics grade, Thermo Fisher) and digested for 6 h at 37°C in a Eppendorf ThermoMixer with agitation at 1,300 rpm. After digestion supernatants were taken to fresh tubes and beads washed once to complete peptide recovery. Digests were dried in a vacuum centrifuge. For TMT labelling, 0.2 μg aliquots of TMT labels were resuspended in 9 μl of acetonitrile and added to the digests which had been resuspended in 21 μl 100 mM TEAB pH 8.5. A portion of the labelled samples were pooled and analysed by LC-MS, these data were used to normalise the pooling prior to further analysis. Final pooled samples were cleaned up by SPE using 50 mg tC18 Sep-Pak Cartridges and dried in a vacuum centrifuge. Sample was then resuspended in 40 μl of 200 mM Ammonium formate pH 10 and transferred to a glass HPLC vial. BpH-RP fractionation was conducted on an Ultimate 3000 UHPLC system (Thermo Scientific) equipped with a 2.1 mm × 15 cm, 1.7μ Kinetex EVO column (Phenomenex). Solvent A was 3% ACN, Solvent B was 100% ACN, solvent C was 200 mM ammonium formate (pH 10). Throughout the analysis solvent C was kept at a constant 10%. The flow rate was 400 μl/min and UV was monitored at 280 nm. Samples were loaded in 90% A for 10 min before a gradient elution of 0–10% B over 10 min (curve 3), 10–34% B over 21 min (curve 5), 34–50% B over 5 mins (curve 5) followed by a 10 min wash with 90% B. 15s (100 μl) fractions were collected throughout the run. Fractions containing peptide (as determined by A280) were recombined across the gradient to preserve orthogonality with on-line low pH RP separation, for example fractions 1, 25, 49, 73, 97 are combined. 12 fractions were generated in this manner. Fractions were then dried in a vacuum centrifuge and stored at -20°C until LC-MS analysis.

### Plasmid construction

See S2 Table for all primer sequences.

A plasmid expressing gRNA for directed CRISPR/Cas9 *Cd97* knock-out mutagenesis was obtained by ligation of duplexed DNA primers (sequences are shown in S2 Table) into lentiCRISPRv2 (Addgene #52961) by ligation following its digestion by Esp3I [42].

DNA sequence encoding full length variant of *Cd97* with 2 C-terminal HA tags was obtained from Thermo Fisher Scientific GeneArt service and cut and ligated into M6P expression lentiviral plasmid using EcoRI and NotI restriction sites, yielding plasmid p*Cd97*-2HA. To reconstitute the *Cd97*^-/-^ CRISPR knock out, *Cd97* gene carrying synonymous mutations in the gRNA complementary region was introduced in p*Cd97*-2HA plasmid using overlapping PCR. Two PCR fragments were obtained using primers EcoRI-CD97wt-F with ovCD97mut-R and ovCD97mut-F with NotI-CD97wt-R. A second PCR was then done with the two amplicons as templates using primers EcoRI-CD97wt-F and NotI-CD97wt-R. The resulting DNA fragment and p*Cd97* were digested with EcoRI and NotI before ligation and transformation in DH5α.

### Transfection and virus production

Lentiviruses for transduction were produced in HEK293ET cells by cotransfection as described earlier [8]. HEK293ET cells were seeded 24 h before cotransfection. DNA transfection procedures were carried out using Lipofectamine 2000 according to the manufacturer’s protocol (Life Technologies). Plasmids and lipofectamine 2000 were combined and incubated in OptiMEM for 5 min at room temperature before being added to cells. The lentiviral expression vector M6P encoding CD97-2HA or CD97mut-2HA and the lentiviral expression vector M4P encoding GFP, GFP-SteD, GFP-SteD^ala6^ or GFP-SteD^ala16^ were cotransfected together with the packaging plasmids VSVG and pMD-GAGPOL [43]. The lentiviral expression vector lentiCRISPRv2 [42], Addgene #52961) was co-transfected together with the packaging plasmids psPAX2 and pMD2.G (gifts from Didier Trono). In both cases, the culture medium was replaced 24 h after transfection and the supernatant containing viruses was collected 48 h after transfection and filtered.

### Generation of stable cell lines

To generate MutuDCs stably expressing CD97-2HA, CD97mut-2HA, CD97^K555R^-2HA or CD97^KK704,705RR^-2HA and/or GFP, GFP-SteD, GFP-SteD^ala6^ or GFP-SteD^ala16^, lentiviruses were added to MutuDCs together with polybrene at 8 μg/ml. At 24 h post-transduction, cells were either selected with puromycin (0.25 μg/ml) or blasticidine or sorted by flow cytometry for the GFP constructs.

### CRISPR/Cas9 targeted mutant construction

gRNA for CD97 (CCGTTCCCTACTTGGACACT) was designed using software available on the Dharmacon website (https://dharmacon.horizondiscovery.com/gene-editing/crispr-cas9/crispr-design-tool/). The gRNA sequences were ligated into a lentiviral plasmid as described above (plasmid construction).

To generate mutants of *Cd97* in MutuDCs, cells were transduced with virus encapsulating lentiCRISPRv2-gRNA (encoding both Cas9 and *Cd97* gRNA). After puromycin selection (0.25 μg/ml), cells with no CD97 surface protein were sorted by FACS and gene inactivation was verified by immunoblotting using polyclonal antibodies against both CD97α and β subunits.

### Immunofluorescence microscopy

Cells seeded onto coverslips were washed with PBS, fixed in 3.7 % paraformaldehyde in PBS for 20 min at 37°C as described earlier [8]. For surface labelling, primary and secondary antibodies were diluted in 10% horse serum (Sigma) and coverslips were washed in PBS. For intracellular labelling, fixed MutuDCs were permeabilised with 0.1% Triton X-100 in 10% horse serum and subsequently washed 3 times with PBS and blocked with 10% horse serum for 1 h at room temperature. Coverslips were incubated with appropriate primary antibodies (see key resources table) for 1 h at room temperature, washed in PBS, then incubated with secondary antibodies and/or phalloidin for 1 h at room temperature. Finally, coverslips were washed 3 times in PBS and twice in dH_2_O and then mounted onto glass slides using Aqua-Poly/Mount (Polysciences). Coverslips were imaged using an LSM 710 inverted confocal laser-scanning microscope (Zeiss GmbH).

### Image analysis

Cell surface and intracellular signals were calculated using CellProfiler. Cells were identified as primary objects using DAPI, MutuDC-inherent GFP or CellTracker Blue signal in B3Z cells. Cell edges were identified by the limit of MutuDC-inherent GFP signal and the IS at the MutuDC-B3Z T cell interface was identified by F-actin staining with phalloidin at the border between GFP^+^ MutuDC and CellTracker Blue^+^ B3Z cells.

Pearson’s correlation coefficient was used to quantify the colocalisation of CD97β-2HA, GFP-SteD and TfR. The extracellular background signal was subtracted from images using the Background Subtraction function in ImageJ, with a rolling ball radius equal to 200 pixels or 26.4 μm. Pearson’s correlation coefficient values were obtained from individual cells using the Coloc 2 ImageJ plugin (http://imagej.net/Coloc_2).

To count the number of interactions between MutuDCs and B3Z T cells, the MutuDC-inherent GFP+ signal and CellTracker Blue signal in B3Z cells were both dilated by 10 pixels in CellProfiler and number of areas with both signals was enumerated. The percentage of MutuDCs interacting with a B3Z T cell was calculated as number of GFP areas with CellTracker Blue signal/total number of MutuDCs * 100.

### Flow cytometry

Surface levels of MHCII and CD97α on MutuDC cells were measured following infection as described above and as described earlier [8]. In brief, MutuDCs were detached from cell culture plates using 2 mM EDTA in PBS. All antibodies were diluted in FACS buffer (2mM EDTA and 2% FCS in PBS or HBSS as appropriate). Cells were labelled with anti-MHCII (I-A/I-E, clone M5/114, APC-conjugated; Miltenyi Biotec; 130-102-898) and anti-CD97α (clone 587702, PE-conjugated) at 1:200 dilution for 30 min on ice and washed in cold PBS. After washing with cold PBS, cells were fixed in 3.7% paraformaldehyde for 1 h at room temperature. Intracellular *S*. Typhimurium was detected by bacterial expression of GFP or mCherry or by labelling using anti-CSA (KPL; 01-91-99) at 1 :400 dilution for 30 min on ice and subsequent donkey anti-goat secondary antibody (Alexa flour 488).

Primary DCs were purified from single-cell suspensions of mesenteric lymph nodes of C57BL/6 mice infected with GFP-expressing *S*. Typhimurium using anti-CD11c antibody-coupled magnetic beads (Miltenyi Biotec; 130-125-835) according to the manufacturer’s instructions. Non-specific antibody binding to purified cells was blocked using FcR blocking reagent (Miltenyi Biotec; 130-092-575). Purified DCs were labelled on ice with anti-CD97α (RandD Systems; FAB3734P) antibody diluted in FACS buffer for 30 min on ice. Purity was assessed by anti-CD11c antibody (clone N418, VioBlue-conjugated; Miltenyi Biotec; 130-102-797) labelling. Discrimination between infected and non-infected cells was based on GFP fluorescence and CD97α median fluorescence calculated as described above.

For analysis of CD69 surface levels on primary T cells, the CD11c^-^ fraction from DC purification was used. T cells were labelled on ice with anti-CD11c, anti-CD3ε (Miltenyi Biotec; 130-102-794), anti-CD4 (BD Biosciences; 550954), anti-CD8 (Miltenyi Biotec; 130-108-882), anti CD69 (BD Biosciences; 563290) antibodies diluted in FACS buffer for 30 min on ice. CD69 positivity was assessed in comparison to CD69 fluorescence minus one (FMO) control.

Data were acquired using a Fortessa flow cytometer (BD Biosciences) and analysed using FlowJo v10 software. Surface levels of proteins of interest were calculated as median fluorescence of infected cells/median fluorescence of non-infected cells.

### Co-immunoprecipitations

WT MutuDCs or MutuDCs expressing CD97-2HA and GFP or GFP-SteD from M4P plasmid were harvested in PBS containing 5 mM EDTA and washed once in PBS. Cells were lysed in lysis buffer (150 mM NaCl, 50 mM Tris pH 7.4, 5 mM EDTA, 0.5% Triton X100, 5% glycerol, 10 mM iodoacetamide and protease inhibitor cocktail tablets (Roche)) for 10 min at 4°C as described earlier [8]. The post-nuclear supernatant was obtained by centrifugation at 16,000 *g* for 15 min at 4°C. Proteins were immunoprecipitated by incubation with anti-HA sepharose beads (Pierce) for 7 h at 4°C. Immunoprecipitates were washed four times with wash buffer (150 mM NaCl, 50 mM Tris pH 7.4, 5 mM EDTA, 0.1% Triton X100, 5% glycerol, 10 mM iodoacetamide and protease inhibitor cocktail tablets (Roche)) and boiled in SDS buffer at 95°C for 5 min before analysis by SDS-PAGE and immunoblotting using anti-CD97α (RandD Systems; MAB3734), anti-GFP (Invitrogen; G10362), anti-transferrin receptor (Zymed; 13–6800), anti-HA (Biolegend; 901502), anti-Tmem127 (Bethyl laboratories; A303-450A), anti-Wwp2 (Abcam; ab103527), anti-actin (Abcam; ab8226) and anti DnaK (ENZO; ADI-SPA-880-F) antibodies. Immunoblots were visualised using ECL detection reagents (GE Healthcare, Thermo Scientific) on a Chemidoc Touch Imaging System (Bio-Rad) and densitometry measurements were carried out using Image Lab software (Bio-Rad).

### Mass spectrometry

To identify ubiquitinated positions in CD97, all results were internally calibrated and searched against a database with the Laboratory of Mass Spectrometry users’ sequences to focus on proteins of interests. Search parameters for precursor and product ions mass tolerance were 8 ppm and 0.1 Da, respectively, enzyme specificity: none and variable modification of methionine oxidation, cysteine methylthio, and lysine ubiquitination (di-glycine tag). Modified peptides were manually inspected.

To analyse the samples obtained by plasma membrane protein biotinylation using Aminooxy-Biotin, the collected protein fractions were analysed on an Orbitrap Fusion instrument on-line with an Ultimate 3000 RSLC nano UHPLC system (Thermo Fisher). Samples were resuspended in 10 μl of 5% DMSO/1% TFA. 5 μl of each fraction was injected. Trapping solvent was 0.1% TFA, analytical solvent A was 0.1% FA, solvent B was ACN with 0.1% FA. Samples were loaded onto a trapping column (300μm x 5mm PepMap cartridge trap (Thermo Fisher)) at 10 μl/min for 5 minutes. Samples were then separated on a 75 cm x 75 μm i.d. 2 μm particle size PepMap C18 column (Thermo Fisher). The gradient was 3–10% B over 10mins, 10–35% B over 155 minutes, 35–45% B over 9 minutes followed by a wash at 95% B for 5 minutes and re-equilibration at 3% B. Eluted peptides were introduced by electrospray to the MS by applying 2.1kV to a stainless steel emitter (5 cm x 30 μm (PepSep)). Mass spectrometer parameters can be found in S8 Fig.

Raw data were searched using MASCOT (Matrix Science) from within Proteome Discoverer V2.2 (Thermo Fisher) against the SwissProt Mouse reference proteome and a database of common contaminants. MS1/2/tolerance was 10 ppm, 0.6 Da and 20 ppm respectively. Carbamidomethylated cysteine, TMT labelled peptide N-termini and Lysines were set as fixed modification, methionine oxidation was set as a variable modification. Trypsin cleavage rules with a maximum of 2 missed cleaves were set. Peptide false discovery rate was estimated using Mascot Percolator and the resulting protein identifications had either a “high” (<1%FDR) or “medium” (<5%FDR) confidence for identification.

### T cell activation assay

MutuDCs (either WT, *Cd97*^*-/-*^ or *Cd97*^*-/-*^ + CD97-2HA) were infected with stationary phase *S*. Typhimurium 12023 + pGcDi (either WT or Δ*steD*::Km; OD_600_ ~ 2.5 to 3.0) at an MOI of 20:1 as described above. 24 h p.i., SIINFEKL peptide (257–264) derived from chicken ovalbumin was added to the final concentration 100 ng/ml and incubated for 1 h at 37°C, 5% CO_2_. After washing with RPMI1640 medium, DCs were co-incubated with B3Z T cells at a 1:2 ratio in RPMI1640. After 18 h of co-incubation, CD69 surface levels on B3Z T cells were assessed by flow cytometry. MutuDCs not treated with SIINFEKL were used as a negative control to set the CD69^+^ gate.

### Conjugation assay

The DC–T cell conjugation assay was done as described earlier [36] with minor modifications. MutuDCs (either WT, *Cd97*^*-/-*^ or *Cd97*^*-/-*^ + CD97-2HA, inherently GFP^+^) were infected with stationary phase *S*. Typhimurium 12023 carrying pFCcGi encoding mCherry under a constitutive promoter (either WT or Δ*steD*::Km; OD_600_ ~ 2.5 to 3.0) at an MOI of 20:1 as described above. MHCI-specific SIINFEKL peptide (257–264) or MHCII-specific ISQAVHAAHAEINEAGR peptide (323–339), both derived from chicken ovalbumin, were added at 24 h p.i. to a final concentration of 100 ng/ml and incubated for 1 h at 37°C, 5% CO_2_. After washing with RPMI1640 medium, MutuDCs were co-incubated with SIINFEKL-specific B3Z T cells or primary CD4^+^ T cells expressing OVA-specific T cell receptor were isolated from cell suspensions of spleens and lymph nodes of OT-II mice by magnetic sorting of CD4^+^ cells (Miltenyi Biotec; 130-104-454) prelabelled with CellTracker Blue (according to the manufacturer’s instructions) at a 1:2 ratio in RPMI1640 for 1 h. The cell suspension was vortexed at 1,000 RPM for 5 s at RT and cell conjugates were fixed by addition of PFA to a final concentration of 3%. DC-T cell conjugates were measured by flow cytometry and calculated as % of CellTracker Blue^+^ events out of all mCherry^+^GFP^+^ events.

### Statistical analysis

Statistical significance was calculated using two-way ANOVA, one-way ANOVA followed by Tukey’s or Dunnett’s multiple comparison test or one- or two- sample T test as indicated in figure legends. All statistical analysis was carried out using GraphPad Prism v9 software except for TMT reported ion intensities. These were outputted to a.CSV file and submitted to the moderated T-test LIMMA within the R environment. LIMMA p-values were corrected for multiple hypothesis testing using the Benjamini-Hochberg method to generate q-values for each comparison.

## Supporting information

S1 Table Raw values of proteins identified in plasma membrane proteomic screens. (XLSX)

S2 Table Primer sequences. (DOCX)

S1 Fig Representative flow cytometry plots. (A) Representative flow cytometry plots showing surface levels of MHCII, CD97α and CD180 in non-transfected (gate A) and transfected (gate B) MutuDCs stably expressing GFP or GFP-SteD. (B) Representative flow cytometry plots showing surface levels of MHCII, CD97α and CD180 in non-infected (gate A) and infected (gate B) MutuDCs infected with WT, *ΔssaV*, Δ*steD or* Δ*steD* + p*steD S*. Typhimurium. (C) Quantification of CD97α surface levels in HEK cells transiently transfected with vectors encoding CD97-2HA and GFP or GFP-SteD. Cells were analysed by flow cytometry and amounts of surface CD97α are shown as a fraction of surface CD97α in CD97-2HA- and GFP-co-expressing cells. Data are from 3 independent experiments and show means ± SD. *** p < 0.001 (Student’s T-test). (D) Bacterial loads in infected mice. C57BL/6 mice were inoculated orally with WT-GFP or Δ*steD*-GFP *S*. Typhimurium. At the indicated time post inoculation, bacterial load in the extracted homogenised tissue was enumerated by plating and CFU counting. Dots represent single animals from one representative out of three independent experiments.
(PDF)

S2 Fig CD97-TfR-SteD colocalization. (A) Representative confocal immunofluorescence microscopy images of GFP-SteD, CD97β-2HA and TfR colocalization in CD97-2HA MutuDCs stably expressing GFP-SteD (white). and activated with 100 ng/ml LPS. Cells were fixed 24 h post-activation, permeabilised with 0.01% TritonX-100 and labelled for CD97α (red), TFR (green) and DAPI (blue). Arrowheads indicate vesicles containing CD97β-2HA, TfR and GFP-SteD. Scale bar—10 μm. (B) CD97α band after HA coimmunoprecipitation corresponds to the largest variant in Input. CD97β-2HA was immunoprecipitated from *Cd97*^*-/-*^ + CD97-2HA MutuDCs using anti-HA antibody. Final HA-IP sample was mixed with the input sample at the indicated ratios and incubated at 95°C for 5 min before loading on an SDS-PAGE gel. Samples were analysed by immunoblot using polyclonal anti-CD97α.
(PDF)

S3 Fig Tmem127 and Wwp2 are important for effect of SteD on CD97. (A) Quantification of CD97α surface levels in WT, *Tmem127*^*-/-*^ or *Wwp2*^-/-^ MutuDCs. Cells were analysed by flow cytometry and amounts of surface CD97α in knockout cells are shown as a percentage of surface CD97α in WT cells. Data are from 3 independent experiments and show means ± SD. * p < 0.05, ** p < 0.01 (one-sample T-test). (B) Representative flow cytometry plots showing surface levels of CD97α in non-infected (gate A) and infected (gate B) WT, *Tmem127*^*-/-*^ or *Wwp2*^-/-^ MutuDCs infected with WT-mCherry, Δ*steD*-mCherry *or* Δ*steD* + p*steD*-mCherry *Salmonella*. (C) Schematic of murine CD97 showing position of the gRNA used to construct MutuDC cell knockout used in all experiments. The extracellular secretion signal peptide, EGF-like domains, autoproteolytic cleavage site, ubiquitinated amino acids and transmembrane domains (TM) are indicated. (D) Total levels of CD97α and β subunits in whole cell lysates of WT, *Cd97*^*-/-*^, *Cd97*^*-/-*^ + CD97-2HA, *Cd97*^*-/-*^ + CD97^K555R^-2HA or *Cd97*^*-/-*^ + CD97^KK704,705RR^-2HA MutuDCs. Samples were analysed by SDS-PAGE and immunoblot using polyclonal anti-CD97α and anti-CD97β antibodies and monoclonal anti-actin antibody. (E) Quantification of CD97α surface levels in WT, *Cd97*^*-/-*^ + CD97-2HA, *Cd97*^*-/-*^ + CD97^K555R^-2HA or *Cd97*^*-/-*^ + CD97^KK704,705RR^-2HA MutuDCs. Cells were analysed by flow cytometry and amounts of surface CD97α are expressed as a fraction of fluorescence of WT MutuDCs. Data are from 3 independent experiments and show means ± SD. NS–not significant (one-way ANOVA followed by Tukey’s multiple comparison test).
(PDF)

S4 Fig SteD inhibits T cell activation *in vivo*. (A) Quantification of CD97α surface levels in CD103^+^CD11b^-^, CD103^+^CD11b^+^, CD103^-^CD11b^+^ DCs *in vivo*. Cells were obtained from draining MLNs of C57BL/6 mice at indicated times post-oral inoculation with WT-GFP or Δ*steD*-GFP *S*. Typhimurium. CD11c^+^ cells were isolated by magnetic separation and CD97α surface levels were analysed by flow cytometry. Amounts of surface CD97α in infected cells are expressed as a fraction of fluorescence of non-infected cells in the same sample. Each dot represents the value from CD11c^+^ cells obtained from MLNs pooled from two or three mice from three independent experiments and means ± SD are shown. ** p < 0.01, *** p < 0.001 (Student’s T-test). (B) Percentage of CD4^+^ and CD8^+^ T cells out of all T cells isolated from MLNs of C57BL/6 mice orally inoculated as in (A). Data represent percentages at 6 days post inoculation. Data are from MLNs obtained from 15 mice from 3 independent experiments and show means ± SD. NS—not significant (Student’s T-test). (C) Representative flow cytometry histograms showing CD69 surface levels on CD4^+^ and CD8^+^ T cells isolated from MLNs at day 6 post oral inoculation of C57BL/6 with indicated *S*. Typhimurium strains.
(PDF)

S5 Fig Effect of SteD on B3Z T cell surface CD69 is CD97-dependent. (A) Quantification of SIINFEKL-loaded MHCI surface levels in MutuDCs stably expressing GFP or GFP-SteD and activated with 100 ng/ml LPS or in MutuDCs infected with WT-GFP or Δ*steD*-GFP *Salmonella*. Cells were analysed by flow cytometry 24 h post activation/infection and 1 h pulse of 100 ng/ml of SIINFEKL and amounts of surface SIINFEKL-loaded MHCI in transfected or infected cells are expressed as a fraction of fluorescence of non-transfected or non-infected cells in the same sample. Data are from 3 independent experiments and show means ± SD. NS—not significant (Student’s T-test). (B) Representative flow cytometry histograms showing CD69 surface levels on B3Z T cells following 18 h co-incubation with SIINFEKL-loaded indicated MutuDC cell lines infected with WT-GFP or Δ*steD*-GFP *Salmonella*.
(PDF)

S6 Fig SteD does not influence F-actin accumulation at the immunological synapse. (A) Quantification of CD97β-2HA and actin localisation to immunological synapse from 3 experiments represented in Fig 6A. The synaptic region was identified by F-actin at the DC-T cell interface and the ratio of CD97β-2HA or F-actin signal at the DC-T cell interface to the total CD97β-2HA signal was calculated using CellProfiler software. Each dot represents the value for one cell. Error bars show mean ± SD. *** p < 0.001 (one-way T-test). (B) Quantification of actin localisation at immunological synapse from 3 experiments represented in Fig 6B. The synaptic region was identified by F-actin at the DC-T cell interface and the ratio of F-actin signal at the DC-T cell interface to the total F-actin signal was calculated using CellProfiler software. Each dot represents the value for one cell. Error bars show mean ± SD. NS—not significant (Student’s T-test). (C) WT-GFP or Δ*steD*-GFP *S*. Typhimurium (green) inside SIINFEKL-loaded *Cd97*^-/-^ + CD97-2HA MutuDCs interacting with CellTracker Blue-loaded B3Z T cells (red). The images on the left show merged images from Fig 6B, the images on the right show X-Z plane section on the dotted line. White asterisks identify the represented bacteria. Scale bar—5 μm.
(PDF)

S7 Fig CD97 stabilises DC-T cell interaction. (A Representative flow cytometry histograms showing CellTracker Blue labelling (B3Z T cells) in mCherry^+^GFP^+^ (infected MutuDCs) gate from samples of B3Z T cells co-incubated for 1 h with SIINFEKL-loaded indicated MutuDC cell lines infected with WT-mCherry or Δ*steD*-mCherry *S*. Typhimurium. (B) Lack of Tmem127 does not influence DC-T cell interactions in absence of infection. WT or *Tmem127*^*-/-*^ MutuDCs (GFP^+^) incubated with SIINFEKL peptide were exposed to CellTracker Blue-labelled B3Z T cells. Percentage of MutuDCs (GFP^+^) and B3Z (CellTracker Blue^+^) double positive conjugates are shown as a fraction of all MutuDCs (GFP^+^ events) for each condition. Data are from 3 independent experiments and show means ± SD. NS–not significant (Student’s T-test).
(PDF)

S8 Fig Detailed mass spectrometry method setup. (PDF)

S1 File Excel spreadsheet containing numerical data for main and supporting information Figure panels: Figs 2A, 2B, 2D, 2E, 3B, 3C, 3F, 4A, 4B, 4D, 4E, 5A-a, 5C, 6C, 7B, 7C, 7E, 7F, 7G, S1C, S1D, S3A, S3E, S4A, S4B, S5A, S6A, S6B and S7B. (XLSX)
