# Supplementary material for: CD97 stabilises the immunological synapse between dendritic cells and T cells and is targeted for degradation by the Salmonella effector SteD
Source: PLoS Pathog. 2021 Jul 27;17(7):e1009771. doi: 10.1371/journal.ppat.1009771 (PMC8345877; doi:10.1371/journal.ppat.1009771)
Supplement: S2 Table — (DOCX) [file ppat.1009771.s002.docx]

**Table S2. Primer sequences**

| **Use** | **Name** | **Sequence (5’ - 3’)** |
| --- | --- | --- |
| Plasmid: lentiCRISPRv2 Cd97 | Cd97KO-F | caccgCCGTTCCCTACTTGGACACT |
|  | Cd97KO-R | aaacAGTGTCCAAGTAGGGAACGGc |
| Plasmid: CD97-2HA | EcoRI-CD97wt-F | ggGAATTCgccaccatggcaATGAGGGGCGTCAGATGCC |
|  | ovCD97mut-R | CACCATATGCCAAGAGCCACCcTTgCCaACaTGGACttTGCTGCCCACAGCCC |
|  | NotI-CD97wt-R | GGGCGGCCGCtcagaggctagcataatcaggaacatcatacggatacgcatagtccggcacatcatacggataCATCCCTGATTCTGAGGACC |
|  | ovCD97mut-F | GGGCTGTGGGCAGCAAAGTCCATGTTGGCAAGGGTGGCTCTTGGCATATGGTG |
| Plasmid: CD97^K555R^-2HA | ovCD97^K555R^-F | CTGCTGGTGaggCCCATCCAGAGCTCTCGAAC |
|  | ovCD97^K555R^-R | CTGGATGGGcctCACCAGCAGGAAGGTCAGG |
| Plasmid: CD97^KK704,705RR^-2HA | ovCD97^KK704,705RR^-F | GAAGCTCACAaggaggTTTTCTGAAATCAACCCAAACATG |
|  | ovCD97^KK704,705RR^-R | GATTTCAGAAAAcctcctTGTGAGCTTCCAGACAGTGATC |
