# Supplementary figures and images for: CD97 stabilises the immunological synapse between dendritic cells and T cells and is targeted for degradation by the Salmonella effector SteD
Source: PLoS Pathog. 2021 Jul 27;17(7):e1009771. doi: 10.1371/journal.ppat.1009771 (PMC8345877; doi:10.1371/journal.ppat.1009771)

# Supplementary Figure 1

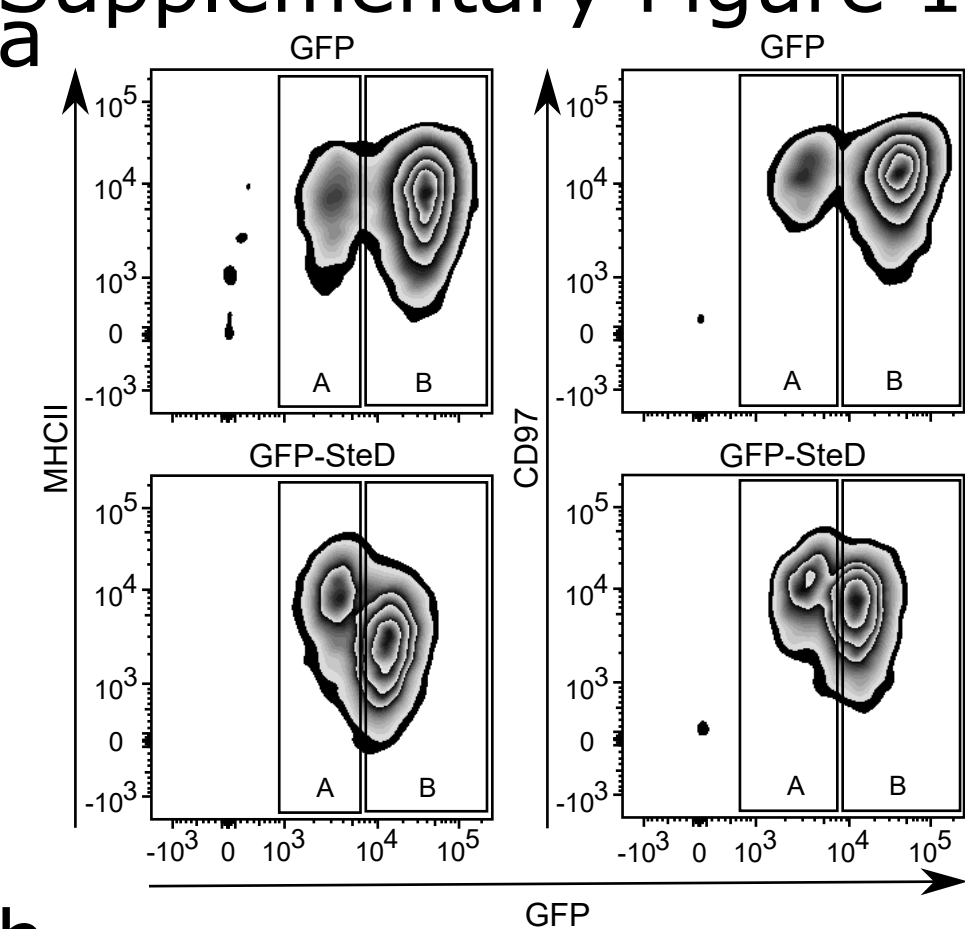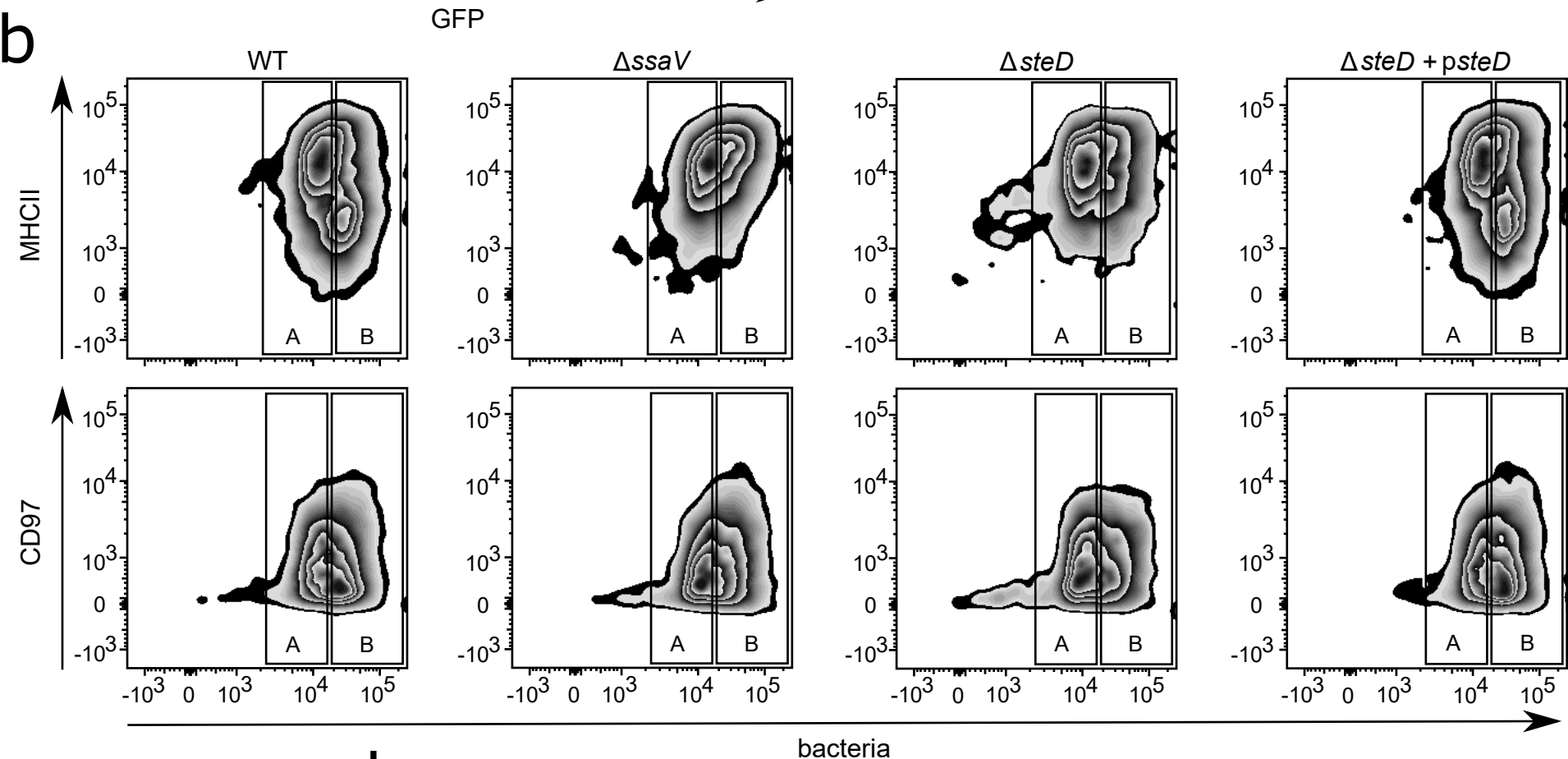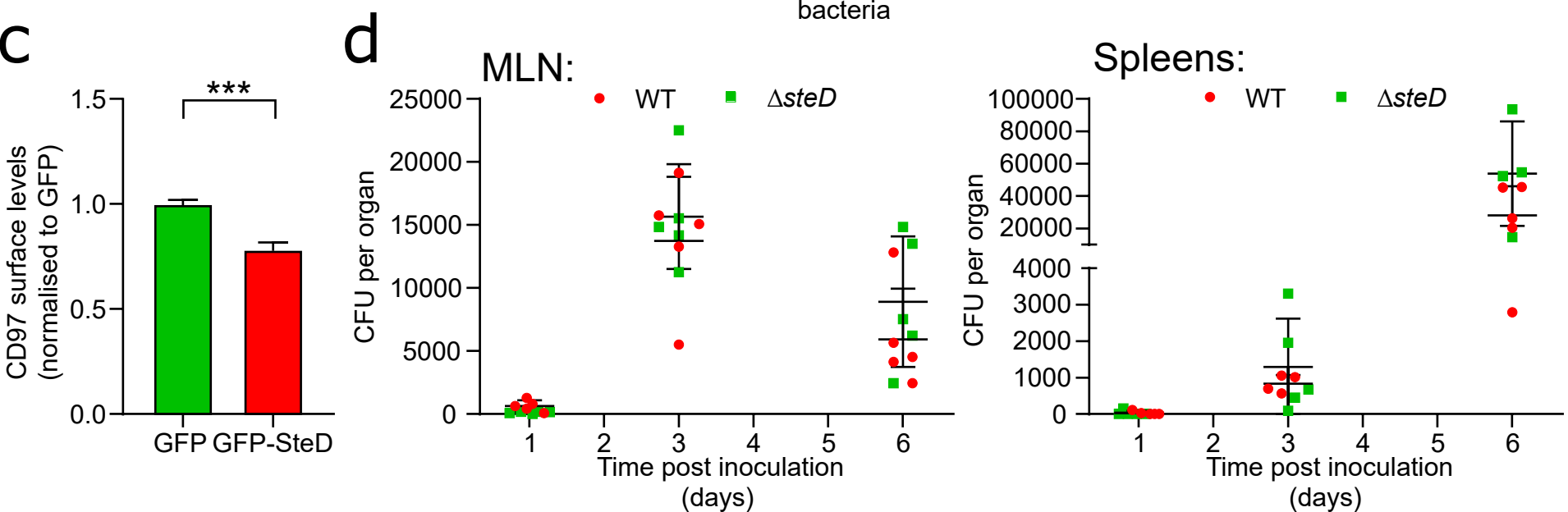

Supplement: S1 Fig — (A) Representative flow cytometry plots showing surface levels of MHCII, CD97α and CD180 in non-transfected (gate A) and transfected (gate B) MutuDCs stably expressing GFP or GFP-SteD. (B) Representative flow cytometry plots showing surface levels of MHCII, CD97α and CD180 in non-infected (gate A) and infected (gate B) MutuDCs infected with WT, ΔssaV, ΔsteD or ΔsteD + psteD S. Typhimurium. (C) Quantification of CD97α surface levels in HEK cells transiently transfected with vectors encoding CD97-2HA and GFP or GFP-SteD. Cells were analysed by flow cytometry and amounts of surface CD97α are shown as a fraction of surface CD97α in CD97-2HA- and GFP-co-expressing cells. Data are from 3 independent experiments and show means ± SD. *** p < 0.001 (Student’s T-test). (D) Bacterial loads in infected mice. C57BL/6 mice were inoculated orally with WT-GFP or ΔsteD-GFP S. Typhimurium. At the indicated time post inoculation, bacterial load in the extracted homogenised tissue was enumerated by plating and CFU counting. Dots represent single animals from one representative out of three independent experiments. (PDF) [file ppat.1009771.s003.pdf]

**A**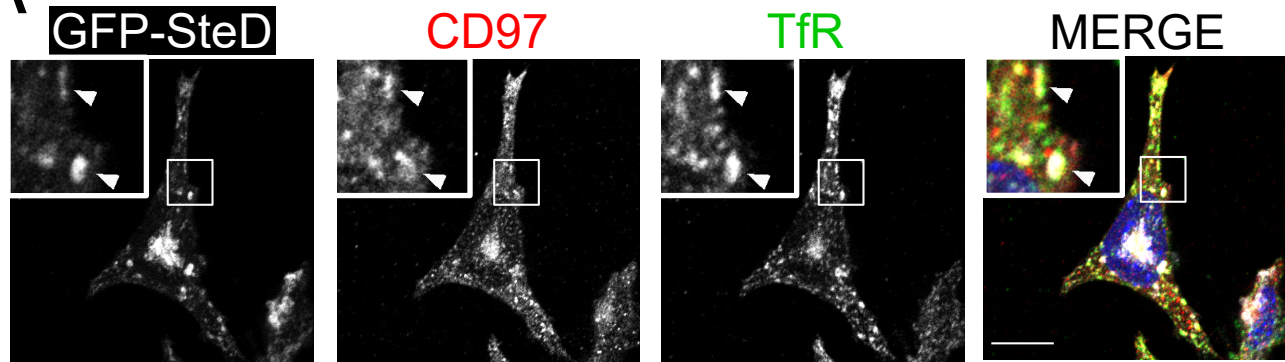**B**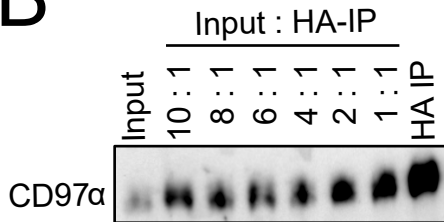

Supplement: S2 Fig — (A) Representative confocal immunofluorescence microscopy images of GFP-SteD, CD97β-2HA and TfR colocalization in CD97-2HA MutuDCs stably expressing GFP-SteD (white). and activated with 100 ng/ml LPS. Cells were fixed 24 h post-activation, permeabilised with 0.01% TritonX-100 and labelled for CD97α (red), TFR (green) and DAPI (blue). Arrowheads indicate vesicles containing CD97β-2HA, TfR and GFP-SteD. Scale bar—10 μm. (B) CD97α band after HA coimmunoprecipitation corresponds to the largest variant in Input. CD97β-2HA was immunoprecipitated from Cd97-/- + CD97-2HA MutuDCs using anti-HA antibody. Final HA-IP sample was mixed with the input sample at the indicated ratios and incubated at 95°C for 5 min before loading on an SDS-PAGE gel. Samples were analysed by immunoblot using polyclonal anti-CD97α. (PDF) [file ppat.1009771.s004.pdf]

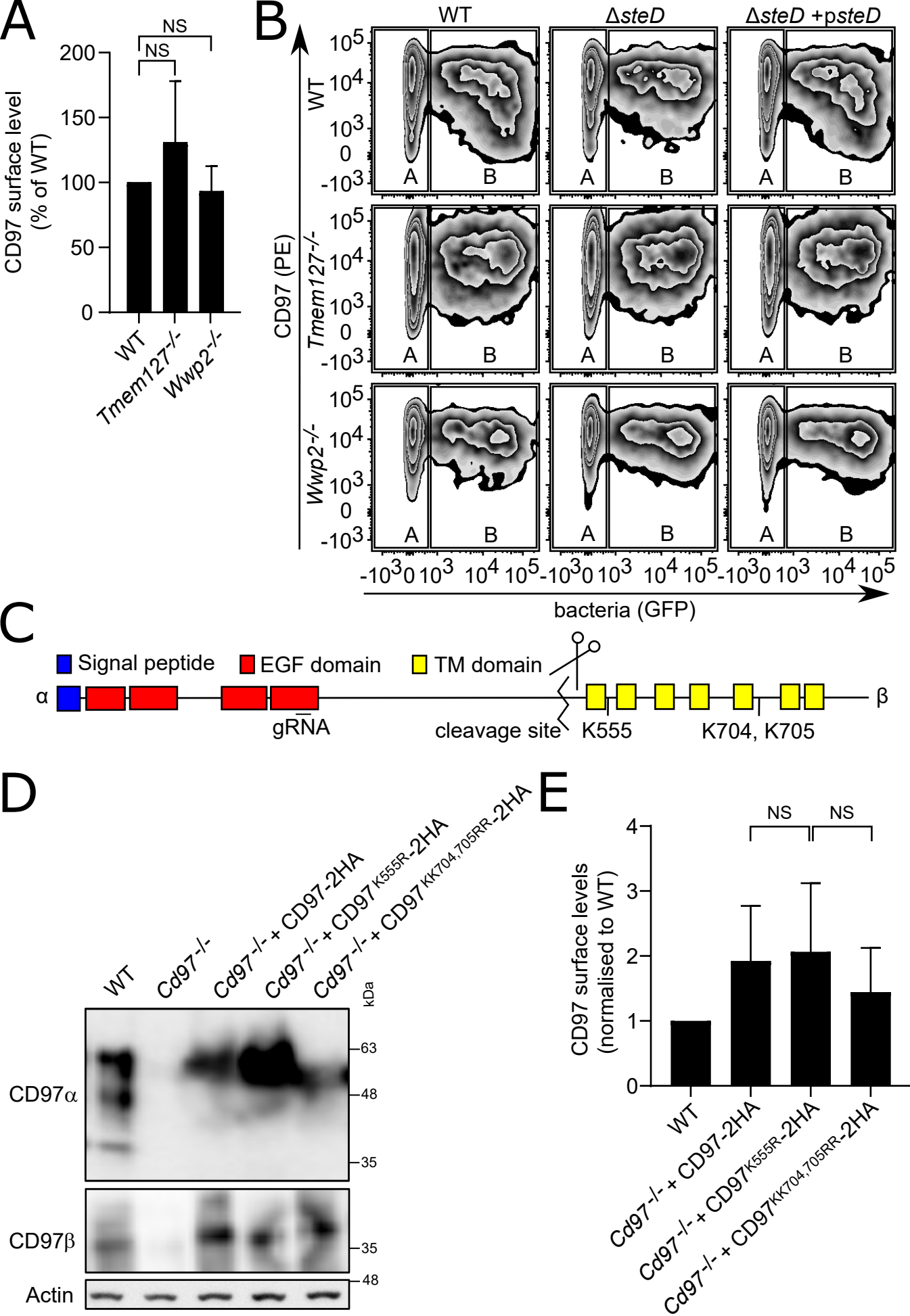

Supplement: S3 Fig — (A) Quantification of CD97α surface levels in WT, Tmem127-/- or Wwp2-/- MutuDCs. Cells were analysed by flow cytometry and amounts of surface CD97α in knockout cells are shown as a percentage of surface CD97α in WT cells. Data are from 3 independent experiments and show means ± SD. * p < 0.05, ** p < 0.01 (one-sample T-test). (B) Representative flow cytometry plots showing surface levels of CD97α in non-infected (gate A) and infected (gate B) WT, Tmem127-/- or Wwp2-/- MutuDCs infected with WT-mCherry, ΔsteD-mCherry or ΔsteD + psteD-mCherry Salmonella. (C) Schematic of murine CD97 showing position of the gRNA used to construct MutuDC cell knockout used in all experiments. The extracellular secretion signal peptide, EGF-like domains, autoproteolytic cleavage site, ubiquitinated amino acids and transmembrane domains (TM) are indicated. (D) Total levels of CD97α and β subunits in whole cell lysates of WT, Cd97-/-, Cd97-/- + CD97-2HA, Cd97-/- + CD97K555R-2HA or Cd97-/- + CD97KK704,705RR-2HA MutuDCs. Samples were analysed by SDS-PAGE and immunoblot using polyclonal anti-CD97α and anti-CD97β antibodies and monoclonal anti-actin antibody. (E) Quantification of CD97α surface levels in WT, Cd97-/- + CD97-2HA, Cd97-/- + CD97K555R-2HA or Cd97-/- + CD97KK704,705RR-2HA MutuDCs. Cells were analysed by flow cytometry and amounts of surface CD97α are expressed as a fraction of fluorescence of WT MutuDCs. Data are from 3 independent experiments and show means ± SD. NS–not significant (one-way ANOVA followed by Tukey’s multiple comparison test). (PDF) [file ppat.1009771.s005.pdf]

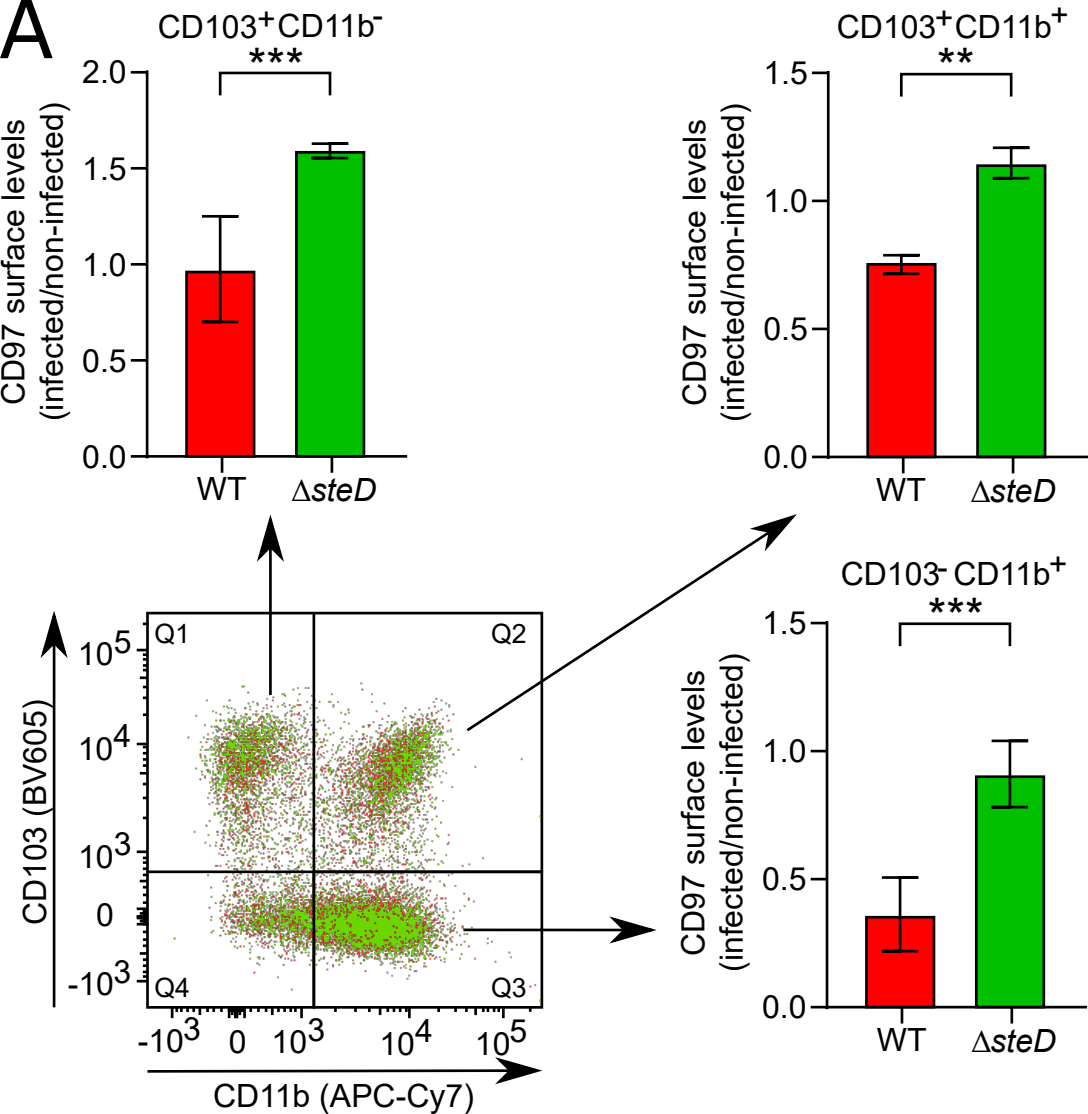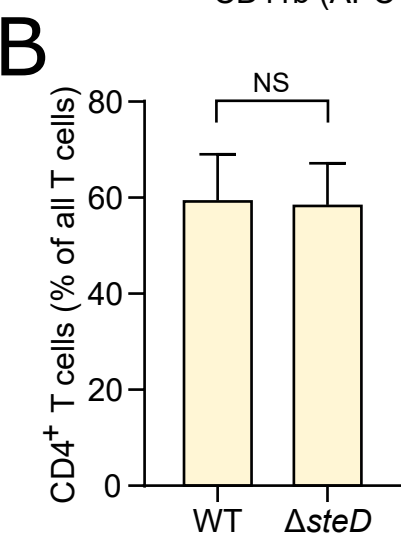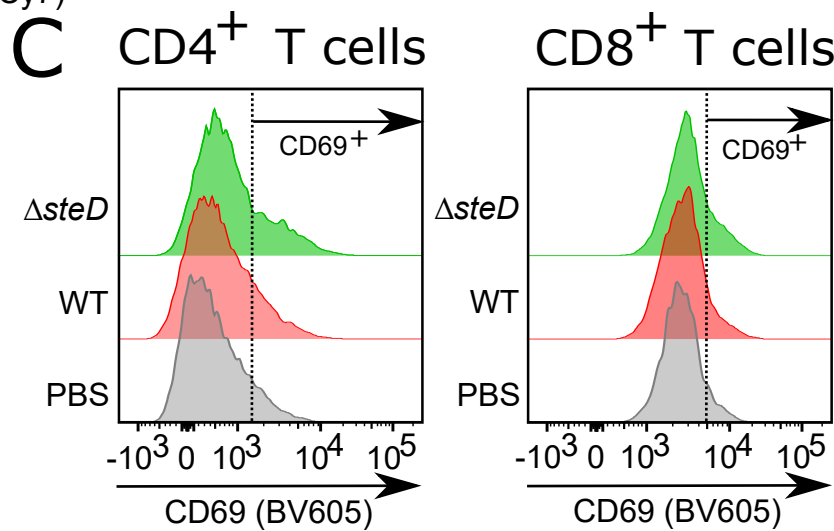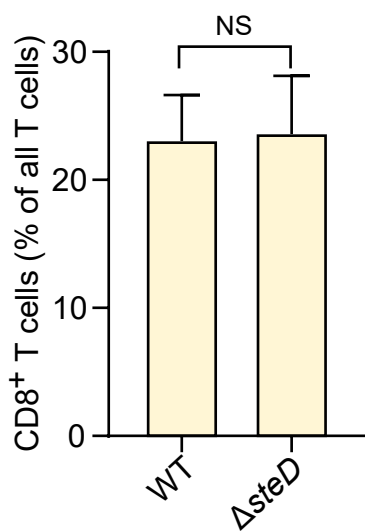

Supplement: S4 Fig — (A) Quantification of CD97α surface levels in CD103+CD11b-, CD103+CD11b+, CD103-CD11b+ DCs in vivo. Cells were obtained from draining MLNs of C57BL/6 mice at indicated times post-oral inoculation with WT-GFP or ΔsteD-GFP S. Typhimurium. CD11c+ cells were isolated by magnetic separation and CD97α surface levels were analysed by flow cytometry. Amounts of surface CD97α in infected cells are expressed as a fraction of fluorescence of non-infected cells in the same sample. Each dot represents the value from CD11c+ cells obtained from MLNs pooled from two or three mice from three independent experiments and means ± SD are shown. ** p < 0.01, *** p < 0.001 (Student’s T-test). (B) Percentage of CD4+ and CD8+ T cells out of all T cells isolated from MLNs of C57BL/6 mice orally inoculated as in (A). Data represent percentages at 6 days post inoculation. Data are from MLNs obtained from 15 mice from 3 independent experiments and show means ± SD. NS—not significant (Student’s T-test). (C) Representative flow cytometry histograms showing CD69 surface levels on CD4+ and CD8+ T cells isolated from MLNs at day 6 post oral inoculation of C57BL/6 with indicated S. Typhimurium strains. (PDF) [file ppat.1009771.s006.pdf]

**A**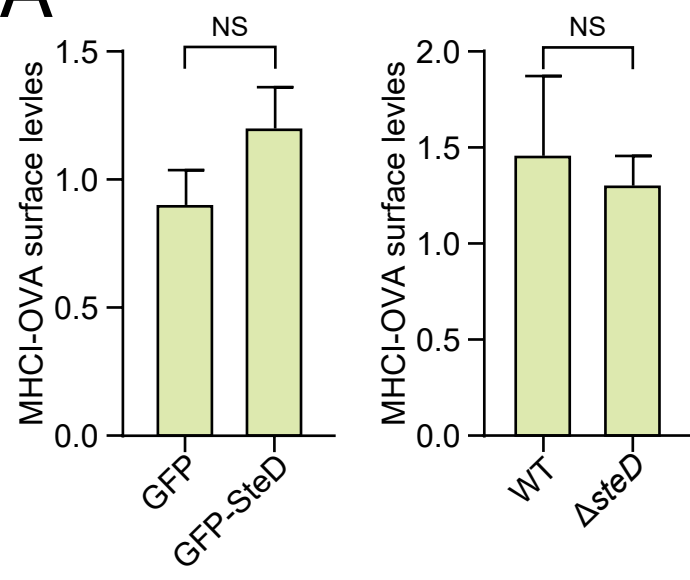**B**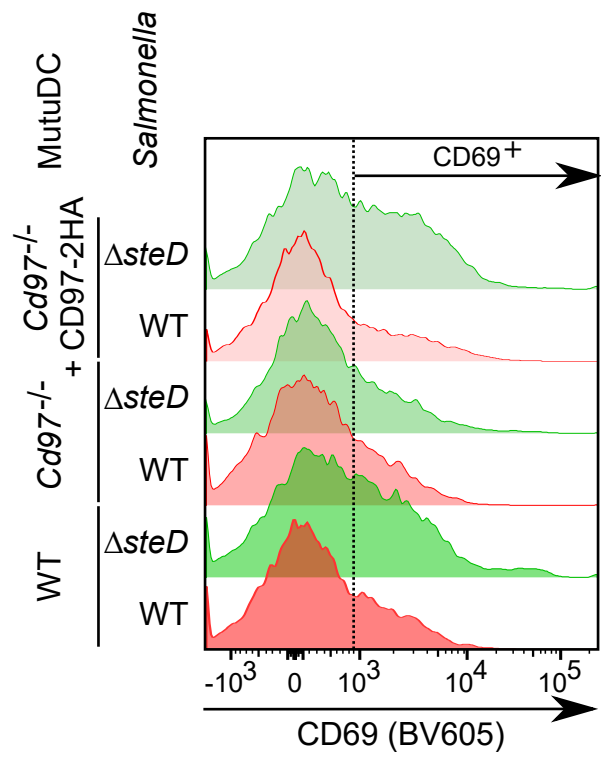

Supplement: S5 Fig — (A) Quantification of SIINFEKL-loaded MHCI surface levels in MutuDCs stably expressing GFP or GFP-SteD and activated with 100 ng/ml LPS or in MutuDCs infected with WT-GFP or ΔsteD-GFP Salmonella. Cells were analysed by flow cytometry 24 h post activation/infection and 1 h pulse of 100 ng/ml of SIINFEKL and amounts of surface SIINFEKL-loaded MHCI in transfected or infected cells are expressed as a fraction of fluorescence of non-transfected or non-infected cells in the same sample. Data are from 3 independent experiments and show means ± SD. NS—not significant (Student’s T-test). (B) Representative flow cytometry histograms showing CD69 surface levels on B3Z T cells following 18 h co-incubation with SIINFEKL-loaded indicated MutuDC cell lines infected with WT-GFP or ΔsteD-GFP Salmonella. (PDF) [file ppat.1009771.s007.pdf]

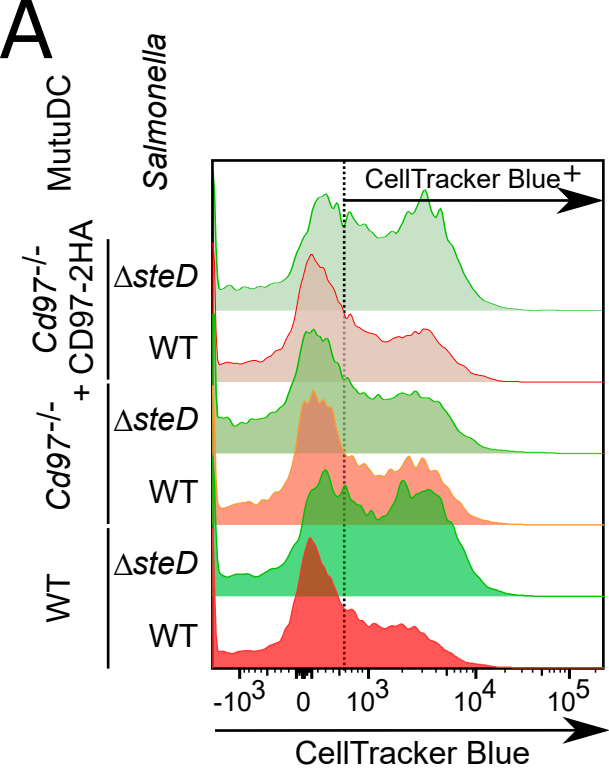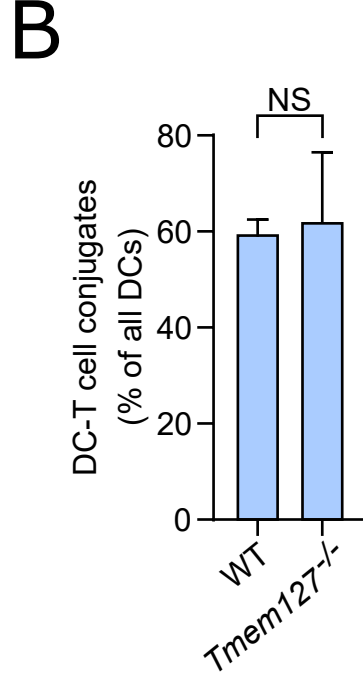

Supplement: S7 Fig — (A Representative flow cytometry histograms showing CellTracker Blue labelling (B3Z T cells) in mCherry+GFP+ (infected MutuDCs) gate from samples of B3Z T cells co-incubated for 1 h with SIINFEKL-loaded indicated MutuDC cell lines infected with WT-mCherry or ΔsteD-mCherry S. Typhimurium. (B) Lack of Tmem127 does not influence DC-T cell interactions in absence of infection. WT or Tmem127-/- MutuDCs (GFP+) incubated with SIINFEKL peptide were exposed to CellTracker Blue-labelled B3Z T cells. Percentage of MutuDCs (GFP+) and B3Z (CellTracker Blue+) double positive conjugates are shown as a fraction of all MutuDCs (GFP+ events) for each condition. Data are from 3 independent experiments and show means ± SD. NS–not significant (Student’s T-test). (PDF) [file ppat.1009771.s009.pdf]
