# Supplementary material for: CD97 stabilises the immunological synapse between dendritic cells and T cells and is targeted for degradation by the Salmonella effector SteD
Source: PLoS Pathog. 2021 Jul 27;17(7):e1009771. doi: 10.1371/journal.ppat.1009771 (PMC8345877; doi:10.1371/journal.ppat.1009771)
Supplement: S6 Fig — (A) Quantification of CD97β-2HA and actin localisation to immunological synapse from 3 experiments represented in Fig 6A. The synaptic region was identified by F-actin at the DC-T cell interface and the ratio of CD97β-2HA or F-actin signal at the DC-T cell interface to the total CD97β-2HA signal was calculated using CellProfiler software. Each dot represents the value for one cell. Error bars show mean ± SD. *** p < 0.001 (one-way T-test). (B) Quantification of actin localisation at immunological synapse from 3 experiments represented in Fig 6B. The synaptic region was identified by F-actin at the DC-T cell interface and the ratio of F-actin signal at the DC-T cell interface to the total F-actin signal was calculated using CellProfiler software. Each dot represents the value for one cell. Error bars show mean ± SD. NS—not significant (Student’s T-test). (C) WT-GFP or ΔsteD-GFP S. Typhimurium (green) inside SIINFEKL-loaded Cd97-/- + CD97-2HA MutuDCs interacting with CellTracker Blue-loaded B3Z T cells (red). The images on the left show merged images from Fig 6B, the images on the right show X-Z plane section on the dotted line. White asterisks identify the represented bacteria. Scale bar—5 μm. (PDF) [file ppat.1009771.s008.pdf]

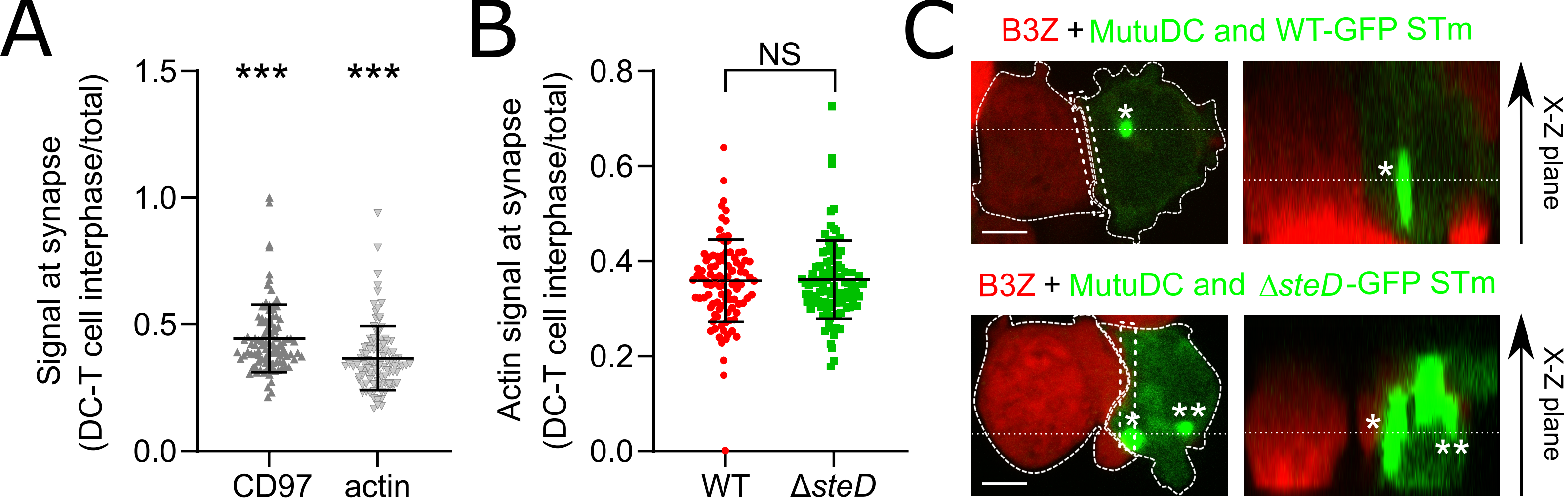
A
Signal at synapse
(DC-T cell interphase/total)
1.5
1.0
0.5
0.0
***
***
CD97
actin
B
Actin signal at synapse
(DC-T cell interphase/total)
0.8
0.6
0.4
0.2
0.0
NS
WT
ΔsteD
C
B3Z + MutuDC and WT-GFP STm
X-Z plane
B3Z + MutuDC and ΔsteD-GFP STm
X-Z plane
*
**
