# Supplementary material for: CD97 stabilises the immunological synapse between dendritic cells and T cells and is targeted for degradation by the Salmonella effector SteD
Source: PLoS Pathog. 2021 Jul 27;17(7):e1009771. doi: 10.1371/journal.ppat.1009771 (PMC8345877; doi:10.1371/journal.ppat.1009771)
Supplement: S8 Fig — (PDF) [file ppat.1009771.s010.pdf]

Start Time (min): **0**
End Time (min): **190**
Cycle Time (sec): **3**

Master Scan:
- MS OT
  - Detector Type: **Orbitrap**
  - Orbitrap Resolution: **120000**
  - Mass Range: **Normal**
  - Use Quadrupole Isolation: **True**
  - Scan Range (m/z): **400-1500**
  - RF Lens (%): **60**
  - AGC Target: **Custom**
  - Normalized AGC Target (%): **125**
  - Maximum Injection Time Mode: **Custom**
  - Maximum Injection Time (ms): **50**
  - Microscans: **1**
  - Data Type: **Profile**
  - Polarity: **Positive**
  - Source Fragmentation: **Disabled**
  - Scan Description:

Filters:
- MIPS
  - Monoisotopic Peak Determination: **Peptide**
- Charge State
  - Include charge state(s): **2-7**
  - Include undetermined charge states: **False**
- Dynamic Exclusion
  - Use Common Settings: **False**
  - Exclude after n times: **1**
  - Exclusion duration (s): **90**
  - Mass Tolerance: **ppm**
  - Low: **10**
  - High: **10**
  - Exclude Isotopes: **True**
  - Perform dependent scan on single charge state per precursor only: **True**
- Intensity
  - Filter Type: **Intensity Threshold**
  - Intensity Threshold: **5.0e3**

- Data Dependent
  - Data Dependent Mode: **Cycle Time**
  - Time between Master Scans (sec): **3**

- Scan Event Type 1:
  - Scan:
  - $ddMS^2$ IT CID
    - Isolation Mode: **Quadrupole**
    - Isolation Window (m/z): **0.7**
    - Isolation Offset: **Off**
    - Activation Type: **CID**
    - Collision Energy Mode: **Fixed**
    - CID Collision Energy (%): **35**
    - CID Activation Time (ms): **10**
    - Activation Q: **0.25**
    - Multistage Activation: **False**
    - Detector Type: **Ion Trap**
    - Ion Trap Scan Rate: **Rapid**
    - Mass Range: **Normal**
    - Scan Range Mode: **Auto**
    - AGC Target: **Custom**
    - Normalized AGC Target (%): **80**
    - Maximum Injection Time Mode: **Auto**
    - Microscans: **1**
    - Data Type: **Centroid**
    - Scan Description:

  Filters:
  - Precursor Selection Range
    - Selection Range Mode: **Mass Range**
    - Mass Range (m/z): **400-2000**
  - Precursor Ion Exclusion
    - Exclusion mass width: **m/z**
    - Low: **19**
    - High: **7**
  - Isobaric Tag Loss Exclusion
    - Reagent: **TMT**

  - Data Dependent
    - Data Dependent Mode: **Scans Per Outcome**

  - Scan Event Type 1:
    - Scan:
    - $ddMS^3$ OT HCD
      - MS^n Level: **3**
      - Synchronous Precursor Selection: **True**
      - Number of SPS Precursors: **10**
      - MS Isolation Window (m/z): **2**
      - MS2 Isolation Window (m/z): **2**
      - Isolation Offset: **Off**
      - Activation Type: **HCD**
      - HCD Collision Energy (%): **65**
      - Detector Type: **Orbitrap**
      - Orbitrap Resolution: **50000**
      - Mass Range: **Normal**
      - Scan Range Mode: **Define m/z range**
      - Scan Range (m/z): **100-1000**
      - AGC Target: **Custom**
      - Normalized AGC Target (%): **40**
      - Maximum Injection Time Mode: **Custom**
      - Maximum Injection Time (ms): **120**
      - Microscans: **1**
      - Data Type: **Profile**
      - Use EASY-IC™: **False**
      - Scan Description:
      - Number of Dependent Scans: **3**
